# Variability in intensive care unit admission among pregnant and postpartum women in Canada: a nationwide population-based observational study

**DOI:** 10.1186/s13054-019-2660-x

**Published:** 2019-11-27

**Authors:** Kazuyoshi Aoyama, Ruxandra Pinto, Joel G. Ray, Andrea D. Hill, Damon C. Scales, Stephen E. Lapinsky, Michelle Hladunewich, Gareth R. Seaward, Robert A. Fowler

**Affiliations:** 1 Department of Anesthesia and Pain Medicine, The Hospital for Sick Children, 555 University Ave, #2211, Toronto, ON M5G 1X8 Canada; 2 Institute of Health Policy, Management and Evaluation, University of Toronto, 4th Floor, 155 College St., Toronto, ON M5T 3M6 Canada; 3 Program in Child Health Evaluative Sciences, SickKids Research Institute, 686 Bay St, Toronto, ON M5G 0A4 Canada; 4 Department of Critical Care Medicine, Sunnybrook Health Science Center, 2075 Bayview Ave., Toronto, ON M4N 3M5 Canada; 5 Keenan Research Centre of the Li Ka Shing Knowledge Institute of St. Michael’s Hospital, 209 Victoria St, Toronto, ON M5B 1T8 Canada; 6 Department of Obstetrics and Gynecology, St. Michael’s Hospital, 30 Bond St, Toronto, ON M5B 1W8 Canada; 7 Department of Critical Care Medicine, Mount Sinai Hospital and University Health Network, 600 University Ave., Toronto, ON M5G 1X5 Canada; 8 Kidney Care Centre, Sunnybrook Health Science Center, 1929 Bayview Ave., Toronto, ON M4G 3E8 Canada; 9 Department of Obstetrics and Gynaecology, Division of Maternal-Fetal Medicine, Mount Sinai Hospital, 700 University Ave., Toronto, ON M5G 1X6 Canada

**Keywords:** Maternal health, Pregnant and postpartum women, Variability, ICU admission, Nationwide population-based study, Multi-level regression models

## Abstract

**Background:**

Pregnancy-related critical illness results in approximately 300,000 deaths globally each year. The objective was to describe the variation in ICU admission and the contribution of patient- and hospital-based factors in ICU admission among acute care hospitals for pregnant and postpartum women in Canada.

**Methods:**

A nationwide cohort study between 2004 and 2015, comprising all pregnant or postpartum women admitted to Canadian hospitals. The primary outcome was ICU admission. Secondary outcomes were severe maternal morbidity (a potentially life-threatening condition) and maternal death (during and within 6 weeks after pregnancy). The proportion of total variability in ICU admission rates due to the differences among hospitals was described using the median odds ratio from multi-level logistic regression models, adjusting for individual hospital clusters.

**Results:**

There were 3,157,248 identifiable pregnancies among women admitted to 342 Canadian hospitals. The overall ICU admission rate was 3.2 per 1000 pregnancies. The rate of severe maternal morbidity was 15.8 per 1000 pregnancies, of which 10% of women were admitted to an ICU. The most common severe maternal morbidity events included postpartum hemorrhage (*n* = 16,364, 0.52%) and sepsis (*n* = 11,557, 0.37%). Of the 195 maternal deaths (6.2 per 100,000 pregnancies), only 130 (67%) were admitted to ICUs. Patients dying in hospital, without admission to ICU, included those with cardiovascular compromise, hemorrhage, and sepsis. For 2 pregnant women with similar characteristics at different hospitals, the average (median) odds of being admitted to ICU was 1.92 in 1 hospital compared to another. Hospitals admitting the fewest number of pregnant patients had the highest incidence of severe maternal morbidity and mortality. Patient-level factors associated with ICU admission were maternal comorbidity index (OR 1.88 per 1 unit increase, 95%CI 1.86–1.99), urban residence (OR 1.09, 95%CI 1.02–1.16), and residing at the lowest income quintile (OR 1.44, 95%CI 1.34–1.55).

**Conclusions:**

Most women who experience severe maternal morbidity are not admitted to an ICU. There exists a wide hospital-level variability in ICU admission, with patients living in urban locations and patients of lowest income levels most likely to be admitted to ICU. Cardiovascular compromise, hemorrhage, and sepsis represent an opportunity for improved patient care and outcomes.

## Introduction

There are approximately 300,000 maternal deaths in the world annually [1], and in a recent population-based study, we have highlighted 18 episodes of severe maternal morbidity (i.e., a potentially life-threatening condition) per 1000 deliveries in Canada, increasing 1.3% annually for the last one decade [2]. However, severe maternal morbidity can often be anticipated according to known maternal risk factors, and outcomes possibly improved with early recognition and timely supportive acute and intensive care [3–8].

Variability in both recognition of critical illness and access to intensive care units (ICUs) influences the risk of death in many critical care syndromes [9, 10]. For example, sociodemographic-based variation and health care provider volume of experience have been shown to be important determinants of outcome for patients with acute lung injury, ischemic cardiac disease, and traumatic injury [11–14]. We therefore aimed to describe variation in care for acutely ill pregnant and postpartum women in Canada—a country with a vast geography and provincially administered health care—and to investigate the relationship between patient factors and hospital-based variability in ICU admission practices and clinical outcomes.

## Methods

### Study design, data sources, and population

We conducted a nationwide population-based cohort study in Canada spanning the years from 2004 to 2015, using data from the Discharge Abstract Database (DAD) of Canadian Institute of Health Information (CIHI), which includes information from every admission to acute care hospitals across Canada, except the province of Quebec, which maintains a separate database.

Patients in this cohort were hospitalized pregnant and postpartum women within 6 weeks after the end of pregnancy, including hospitalized women with abortion or ectopic pregnancy. Available variables from the DAD included the following: age, DAD-derived variables (socioeconomic status, comorbid conditions as classified by the International Classification of Diseases and Related Health Problems-Tenth Revision, Canada [ICD-10-CA], interventions and procedures occurring in hospital as determined by the Canadian Classification of Health Interventions [CCI]), pregnancy-specific variables, hospital-level variables, and patient outcomes such as ICU admission, vital status at hospital discharge, and events allowing determination of severe maternal morbidity (Additional file 1: Table S1).

The dataset (the DAD of CIHI) has been well-validated in a number of prior studies [15–20]. In addition, the institute annually reports data quality documentation, to have transparency of data quality. An *episode* of pregnancy was created as previously described [2], so that all related admissions over one pregnancy course could be summarized into one pregnancy episode.

### Outcomes

The primary outcome was admission to ICU which included all admissions to CIHI-derived intensive (special) care units, including “step-up”/“step-down” ICUs [21]. Secondary outcomes included a modified Canadian definition of severe maternal morbidity and maternal death [22, 23]. In order to improve the specificity of the outcome of severe maternal morbidity for this analysis, the definition was modified to exclude events consisting only of a blood transfusion and diagnoses reflecting only *pre-existing* conditions such as HIV, chronic congestive failure, chronic hypertension, and those with an *admission-related* (as opposed to outcome-related) specific diagnosis [e.g., asthma, placenta previa, severe pre-eclampsia, or sickle cell anemia] [2, 8, 22]. Maternal death was defined as death occurring during pregnancy or within 6 weeks after the end of the pregnancy.

### Predictors

A hospital-level predictor was created to represent pregnancy-related admission volumes at each hospital. First, hospitals with fewer than 55 deliveries over the study period (approximately corresponding to fewer than 5 deliveries per year) were excluded using a commonly applied principle of limiting analyses and inferences about data containing fewer than 5 observations in a particular time period (in this case, 1 year). Next, hospital quintiles were created according to pregnancy admission volume at each hospital. Other ordinal predictors (e.g., 5 groups according to ICU admission rate and 5 groups according to the annual number of pregnancy admissions at each hospital) were created in order to perform hospital-related sensitivity analyses.

Comorbid conditions necessary for determining the maternal comorbidity index (MCI) were recorded for each patient, as we have previously determined it to be the most valid risk adjustment tool using health administrative data for hospitalized pregnant and postpartum women [24, 25]. The index ranges from 0 to 45, excluding the age-related variable. Age was removed from the index because the age of the index does not include age younger than 35 years, but age and the index were separately entered into the model. Since six conditions of the maternal comorbidity index had potential overlap with severe maternal morbidity outcome conditions, only the pre-existing conditions were included in the maternal comorbidity index, and those occurring after pregnancy onset were considered as outcomes for severe maternal morbidity, to ensure a valid temporal relation [2].

### Statistical analyses

We used the mean and standard deviation or median and interquartile range to summarize continuous variables, and counts and proportions to describe categorical variables, as appropriate. Standardized differences were employed to compare those who required ICU admission with those who did not, where 0.2, 0.4, and 0.6 standardized differences were generally equated to small, medium, and large effect sizes, respectively [26]. All variables were screened on the basis of standardized difference and selected for regression models if the standardized difference was greater than 0.1, in addition, to an a priori decision to include the urban or rural location of patient residence and hospital location. To develop random intercept logistic regression models with patient and hospital levels, first, we explored an intercept-only model predicting the outcome of ICU admission with each hospital considered as a cluster (representing differences in ICU admission rates among various hospitals). Then, we added patient-level variables to the intercept-only model and finally hospital-level variables [27, 28].

We used the median odds ratio (MOR) to represent the variability of the outcome (e.g., ICU admission) among the hospital clusters of interest [27, 29]. For example, when the MOR = 2, it means that for two pregnant women with the same characteristics, the odds of being admitted to ICU may be twofold higher in one randomly selected hospital compared to another randomly selected hospital from a different cluster when the clusters were ordered by risk. The MOR was computed from the following equation: $\mathrm{MOR}=\exp \Big(0.675\times \sqrt{2\times \mathrm{hospital}-\mathrm{level}\kern0.34em \mathrm{variance}}$, where the hospital-level variance of the parameter estimate (i.e., *β*) is derived from the random intercept model. Interval odds ratio-80 (IOR-80) was used to quantify the impact of a variable at the hospital cluster-level on the outcome (e.g., ICU admission) by incorporating between-cluster variability [27, 29]. IOR-80 covers 80% of the distribution of the odds when comparing two subjects with the same value for all other covariates, but in two different hospitals where hospital-level characteristics may differ. We calculated the lower and upper bounds of the IOR-80 from the following equations: ${\mathrm{IOR}}_{\mathrm{lower}}=\exp \left(\beta -1.2816\times \sqrt{2\times \mathrm{hospital}-\mathrm{level}\kern0.17em \mathrm{variance}}\right)$ and $\mathrm{IO}{\mathrm{R}}_{\mathrm{upper}}=\exp \left(\beta +1.2816\times \sqrt{2\times \mathrm{hospital}-\mathrm{level}\kern0.17em \mathrm{variance}}\right)$, where *β* is the regression coefficient for the hospital level. Note that the IOR does not describe the interval around a MOR; instead, the IOR reflects the size and variation of the odds ratios at the hospital level. If the IOR-80 is wide, it implies high variability in the outcome among hospitals compared to the effect of the cluster level variable. If the interval of IOR-80 crosses 1, it infers that a variable at the hospital cluster level is likely not significantly associated with the outcome. Finally, the variance partitioning coefficient (VPC) represents the proportion of total variability in the outcome (e.g., ICU admission rates) due to the differences among hospital clusters. For multi-level logistic regression models, VPC is computed from the following equation: VPC = hospital-level variance/(hospital-level variance + 3.29) [30].

### Sensitivity analyses

Two pre-planned sensitivity analyses were conducted. First, we explored the association of hospital volume of pregnancy-related admissions as a hospital-level ordinal variable comprised of five categories, on the outcome of ICU admission. In the second sensitivity analysis, we restricted the primary outcome of ICU admission to only admissions to the highest acuity ICUs, excluding step-up/down units.

The third, post hoc, sensitivity analysis was performed where all analyses were redone using multiple imputation datasets, given the rate of missing data in variables reflecting socioeconomic status (Table 1). Missing variables were assessed based on an algorithm reported elsewhere, to determine characteristics of missingness (i.e., *missing completely at random*, *missing at random*, or *missing not at random*) [31]. We used listwise deletion (entire record excluded from analysis if any single value is missing) for the primary analysis. This method is valid if the data is *missing completely at random* [32]. As a sensitivity analysis, to investigate the influence of data *missing not completely at random*, we performed multiple imputation using the fully conditional method with 20 imputed datasets [33, 34]. We imputed missing variables using a fully conditional specification and the discriminant function method to impute the categorical variables: income quintile (14.0% missing), residence rurality (0.7% missing), and hospital rurality (0.6% missing). We created the 20 multiply imputed datasets as this generally provides good efficiency of the parameters (i.e., smaller standard error of the point estimates), in addition to sufficient power to detect differences [35–37].

**Table 1:** General characteristics of pregnant and postpartum women according to whether admitted to ICU or not

	Pregnancies with ICU admission* (*n* = 10,204)	Pregnancies without ICU admission* (*n* = 3,152,099)	Standardized difference^†^
Delivery admissions	9001 (88.2%)	3,020,112 (95.8%)	− 0.28
Antepartum admissions	2596 (25.4%)	250,372 (7.9%)	0.48
Postpartum admissions	2583 (25.3%)	58,652 (1.9%)	0.73
Abortions or ectopic pregnancy-related admissions	918 (9.0%)	107,097 (3.4%)	0.23
Age (years) (mean ± SD)	30.5 ± 6.3	29.5 ± 5.6	0.16
< 15	8 (0.08%)	1191 (0.04%)	0.22
15–19	475 (4.7%)	136,868 (4.3%)	
20–24	1365 (13.5%)	480,141 (15.3%)	
25–29	2491 (24.6%)	913,371 (29.0%)	
30–34	3042 (30.0%)	1,006,926 (32.0%)	
35–39	2026 (20.0%)	500,255 (15.9%)	
40–44	653 (6.4%)	102,423 (3.2%)	
45+	81 (0.8%)	5932 (0.2%)	
Gestational week at delivery admission (weeks)	36.0 ± 4.7	38.7 ± 2.5	− 0.72
Maternal comorbidity index	1.62 ± 2.06	0.47 ± 0.84	0.73
Live birth	9005 (88.2%)	3,020,305 (95.8%)	− 0.28
Singleton birth	8535 (83.6%)	2,970,321 (94.2%)	− 0.34
Twin birth	484 (4.7%)	51,029 (1.6%)	0.18
> Triplet birth	17 (0.2%)	1780 (0.06%)	0.03
Parity			0.17
0	6598 (64.7%)	1,829,634 (58.0%)	
1	1939 (19.0%)	818,031 (26.0%)	
2+	1667 (16.3%)	504,434 (16.0%)	
Residence rurality (0.7% missing)			- 0.01
Urban	8225 (81.2%)	2,559,659 (81.8%)	
Rural	1907 (18.8%)	568,117 (18.2%)	
Residence income quintile (14.0% missing)			0.11
Quintile 1 (lowest)	2603 (29.3%)	684,917 (25.3%)	
Quintile 2	1929 (21.7%)	571,796 (21.1%)	
Quintile 3	1659 (18.7%)	523,475 (19.3%)	
Quintile 4	1485 (16.7%)	493,408 (18.2%)	
Quintile 5 (highest)	1212 (13.6%)	435,658 (16.1%)	
Hospital rurality (0.6% missing)			− 0.01
Urban	9917 (97.8%)	3,066,345 (97.9%)	
Rural	224 (2.2%)	66,429 (2.1%)	
Delivery mode			
Vaginal	2148 (21.0%)	2,157,286 (68.4%)	− 1.08
Cesarean section	4425 (43.4%)	817,172 (25.9%)	
Transfer to another institution for acute care	1594 (15.6%)	38,350 (1.2%)	0.54
Readmission	1381 (13.5%)	42,338 (1.3%)	0.48
Province or territory of admission			
Newfoundland and Labrador	260 (2.5%)	51,481 (1.6%)	0.25
Prince Edward Island	39 (0.4%)	15,410 (0.5%)	
Nova Scotia	226 (2.2%)	95,713 (3.0%)	
New Brunswick	298 (2.9%)	80,163 (2.5%)	
Ontario	5967 (58.5%)	1,524,080 (48.3%)	
Manitoba	376 (3.7%)	181,367 (5.7%)	
Saskatchewan	494 (4.8%)	158,537 (5.0%)	
Alberta	1177 (11.5%)	553,741 (17.6%)	
British Columbia	1308 (12.8%)	473,437 (15.0%)	
Territories	59 (0.6%)	18,170 (0.6%)	
Severe maternal morbidity (any versus none)	5017 (49.2%)	44,995 (1.4%)	1.31
0 (number of severe maternal morbidity events)	4588 (45.0%)	3,106,352 (98.5%)	1.48
1	2616 (25.6%)	41,884 (1.3%)	
2	1351 (13.2%)	2890 (0.09%)	
3	719 (7.0%)	625 (0.02%)	
≥ 4	930 (9.1%)	348 (0.01%)	
Length of hospital stay (days)	11.1 ± 16.4	2.7 ± 3.3	0.71
Hospital mortality during episode of pregnancy care	130 (1.3%)	65 (0.00%)	0.16
Hospital group according to hospital volume of pregnancy			
1 (lowest volume)	204 (2.0%)	19,476 (0.6%)	0.15
2	203 (2.0%)	66,852 (2.1%)	
3	818 (8.0%)	211,392 (6.7%)	
4	2165 (21.2%)	598,849 (19.0%)	
5 (highest)	6814 (66.8%)	2,255,530 (71.6%)	

*Data are presented as mean ± SD, median [interquartiles] or %

^†^Standardized difference = difference in means or proportions divided by standard error. Standardized mean differences of 0.2, 0.5, and 0.8 are often/generally equated to effect sizes of small, medium, and large, respectively

All analyses were performed using SAS statistical software, version 9.4 (SAS Institute Inc., Cary, NC) and Excel for Macintosh, version 15.3.9 (Microsoft Corporation, Redmond, WA). All analyses were completed on September 13, 2018. This study was conducted under the data security and privacy policy of CIHI and approved by the institutional review board at Mount Sinai Hospital in Toronto, Canada (10-0305-C) and University of Toronto (#34468).

## Results

### Incidence and clinical characteristics of pregnancy-related ICU admissions in Canada

**Fig. 1:**
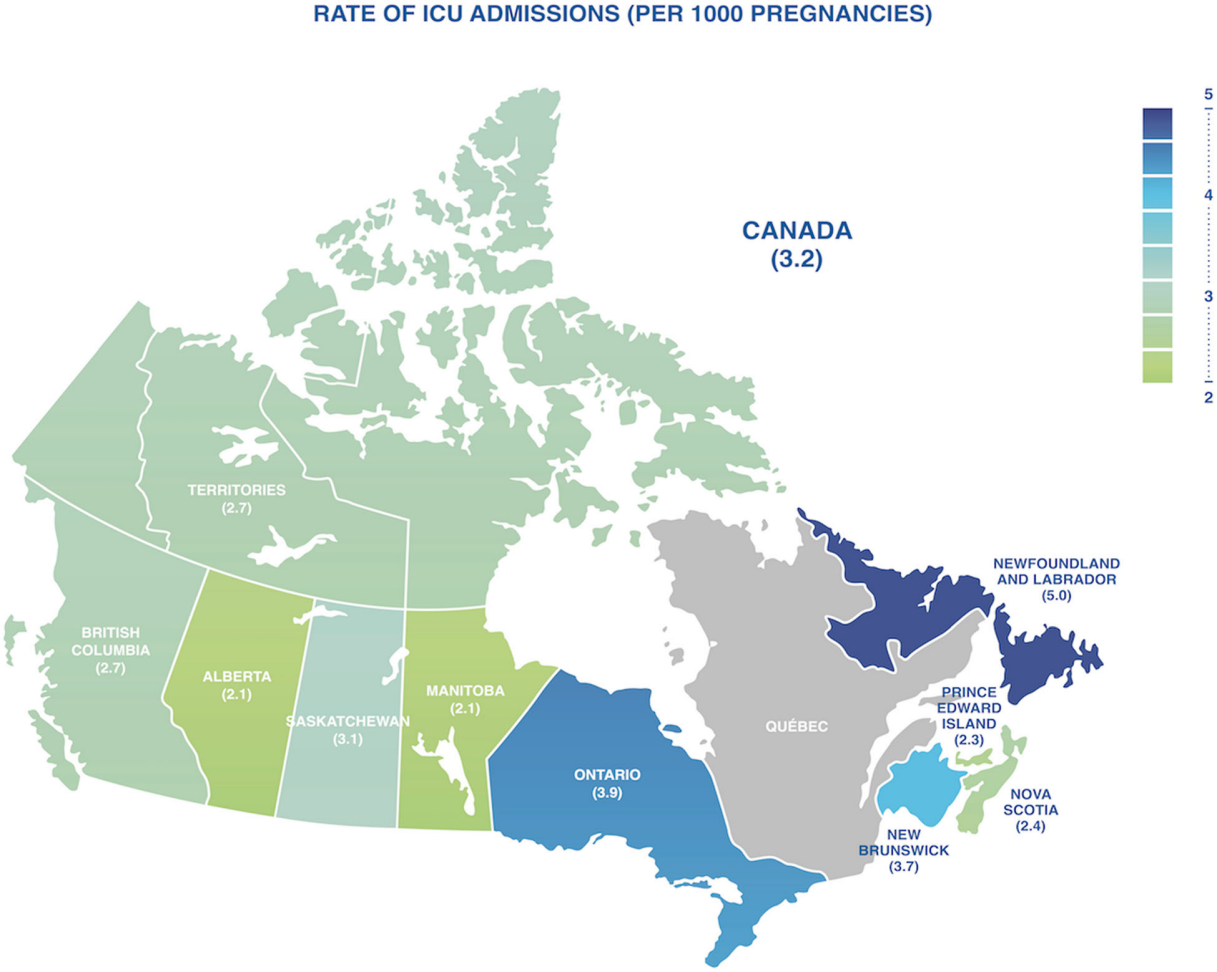
Incidence of ICU admission by province in Canada. Figure legend: territories cover Yukon, Northwest Territories, and Nunavut

During the study period, there were 3,162,303 pregnancies among 2,035,453 mothers, resulting in 10,204 ICU admissions (3.2 cases per 1000 pregnancies) with regional variation (Fig. 1, Table 1). There were 50,012 severe maternal morbidity events (15.8 women with a severe maternal morbidity event per 1000 pregnancies), with 5017 (0.16% overall and 10.0% of those with severe maternal morbidity) pregnant women admitted to ICU (Tables 1 and 2.). A minority of patients experiencing severe maternal morbidity were admitted to ICU (Additional file 2: Table S2, Additional file 3: Table S3). During the study period, there were 195 maternal deaths (6.2 per 100,000 pregnancies (maternal mortality rate 0.01%)); however, only 130 (67%) of these patients were admitted to an ICU prior to death (Table 2). After excluding hospitals with, on average, fewer than 5 deliveries per year, the study cohort consisted of 3,157,248 pregnancies among 342 hospitals (Additional file 4: Table S4, Additional file 5: Table S5).

**Table 2:** Severe maternal morbidity diagnoses: all patients with severe maternal morbidity, those admitted to ICU, and those dying

Individual indicator of several maternal morbidity	No. (%) of patients with the individual severe maternal morbidity indicator among the 50,012 severe maternal morbidity events	No. (%) of patients with an ICU admission with the individual severe maternal morbidity indicator	No. (%) of deaths among patients with the individual severe maternal morbidity indicator
Postpartum hemorrhage or transfusion	16,364 (0.52)	1991 (12.71)	42 (0.26)
Sepsis	11,557 (0.37)	548 (4.74)	21 (0.18)
Myocardial infarction, failure, arrest, or pulmonary edema	4483 (0.14)	1041 (23.22)	101 (2.25)
Placental or uterine abruption and hemorrhage	3758 (0.12)	285 (7.58)	12 (0.32)
Embolization, ligation of pelvic or uterine vessels due to hemorrhage	3015 (0.10)	593 (19.67)	6 (0.20)
Subtotal open hysterectomy	2868 (0.09)	347 (12.10)	11 (0.38)
Eclampsia	2170 (0.07)	467 (21.52)	3 (0.14)
Mechanical ventilation	2099 (0.07)	1933 (92.09)	115 (5.48)
Repair of the bladder, urethra, or intestine	2052 (0.06)	64 (3.12)	1 (0.05)
Hysterectomy and postpartum hemorrhage	1561 (0.05)	728 (46.64)	16 (1.02)
Obstetric embolism	1404 (0.04)	268 (19.09)	34 (2.42)
Placenta previa with hemorrhage or blood transfusion	1343 (0.04)	238 (17.72)	4 (0.30)
Acute renal failure	1116 (0.04)	443 (39.70)	45 (4.03)
Obstetric shock	1124 (0.04)	533 (47.42)	38 (3.38)
Cardiomyopathy in the puerperium	1058 (0.03)	395 (37.33)	9 (0.85)
Evacuation of incisional hematoma	1037 (0.03)	47 (4.53)	0 (0)
Total open hysterectomy	822 (0.03)	387 (47.08)	13 (1.58)
Acute abdomen	790 (0.02)	77 (9.75)	3 (0.38)
Intrapartum hemorrhage and blood transfusion	736 (0.02)	135 (18.34)	7 (0.95)
Cesarean hysterectomy	724 (0.02)	186 (25.69)	4 (0.55)
Acute psychosis	694 (0.02)	16 (2.31)	0 (0)
Pulmonary, cardiac, and central nervous system complications of anesthesia	390 (0.01)	67 (17.18)	3 (0.77)
Cerebrovascular diseases: subarachnoid and intracranial hemorrhage, cerebral infarction, stroke	331 (0.01)	166 (50.15)	30 (9.06)
Disseminated intravascular coagulation	281 (0.01)	192 (68.33)	14 (4.98)
Adult respiratory distress syndrome	252 (0.01)	192 (76.19)	12 (4.76)
Dialysis	202 (0.01)	128 (63.37)	22 (10.89)
Hepatic failure	114 (0.00)	53 (46.49)	14 (12.28)
Cerebral venous thrombosis	111 (0.01)	29 (26.13)	3 (2.70)
Sickle cell anemia with crisis	111 (0.01)	8 (7.21)	0 (0)
Status epilepticus	101 (0.00)	43 (42.57)	0 (0)
Status asthmaticus	76 (0.00)	6 (7.89)	1 (1.32)
Coma	64 (0.00)	37 (57.81)	6 (9.38)
Death^†^	41 (0.00)	8 (19.51)	16 (39.02)

Categories are not mutually exclusive

^†^Overall number of death (any causes) was 195 (0.01%), and 130 patients (66.67%) were admitted to ICU

Patients admitted to an ICU had a higher pre-existing maternal comorbidity index and a resulting greater burden of severe maternal morbidity (Table 1). Among all severe maternal morbidity events, postpartum hemorrhage requiring transfusion and sepsis were the most common (Table 2). Among patients admitted with acute respiratory distress syndrome, cerebral edema or coma, eclampsia, or sepsis, only 76.2%, 57.8%, 21.5%, and 4.7%, respectively, were admitted to ICU. Patients dying in hospital, without admission to ICU, included among others those with some form of cardiovascular compromise (56 patients: obstetric embolism, myocardial insufficiency, or shock), hemorrhage (8 patients), and sepsis (3 patients) (Additional file 6: Table S6).

### Hospital volume of pregnancy admission

We created 5 hospital groupings according to the volume of pregnancy admissions during the study period. Hospitals admitting the fewest number of pregnant patients had the highest incidence of severe maternal morbidity (20 cases per 1000 pregnancies) and had the highest maternal mortality (40 cases per 100,000 pregnancies) (Additional file 4: Table S4), despite having similar pre-existing comorbidities. Hospitals admitting the fewest number of pregnant patients were also more likely to be in rural areas and had a higher proportion of patients in lower income quintiles. There were geographic differences in the proportion of pregnancies admitted at low-to-high-volume hospitals, with Ontario having the greatest proportion of pregnant women admitted to high-volume hospitals (Additional file 4: Table S4, Additional file 5: Table S5).

### Multi-level logistic regression models

The following were entered into the multi-level logistic regression models: patient-level variables (age, maternal comorbidity index, parity, residence location, transfer status for admission) and hospital-level variables (pregnancy-related admission volume, province and territories, hospital location) (Table 3, Additional file 1: Table S1, Additional file 7: Table S7). Independent patient-level factors associated with ICU admission included maternal comorbidity index (1.88, 95%CI 1.86–1.99), urban residence (1.09, 95%CI 1.02–1.16), and lower incomes. The lower income they had, the higher risk of admitting to ICU (i.e., the lowest income had the highest risk of ICU admission: 1.43 with 95%CI 1.33–1.54). Hospital-level factors associated with ICU admission included specific provinces (Table 3, Additional file 1: Table S1, Additional file 7: Table S7).

**Table 3:** Estimated odds ratios for multi-level logistic regression model for intensive care unit admission

Variable	Odds ratio (95%CI)
Patient variables	
Maternal comorbidity index	1.88 (1.86–1.99)
Age (years)	
< 15	1.73 (0.76–3.91)
15–19	1.11 (1.00–1.25)
20–24	Reference
25–29	1.04 (0.97–1.12)
30–34	1.19 (1.11–1.28)
35–39	1.49 (1.38–1.61)
40–44	2.22 (2.00–2.48)
> 44	2.91 (2.24–3.78)
Parity	0.78 (0.76–0.81)
Residence (urban/rural)	1.09 (1.02–1.16)
Transfer between hospitals	13.94 (12.1–13.83)
Income quintile	
1 (lowest)	1.44 (1.34–1.55)
2	1.28 (1.20–1.39)
3	1.20 (1.10–1.28)
4	1.12 (1.03–1.21)
5 (highest)	Reference
Hospital variables	
Hospital (urban versus rural)	1.30 (0.94–1.79)
Groups according to hospital volume of pregnancy	
1 (lowest volume)	12.94 (12.10–13.83)
2	Reference
3	1.36 (1.00–1.86)
4	1.51 (1.10–2.07)
5 (highest volume)	1.32 (0.96–1.80)
Province	
Newfoundland and Labrador	1.20 (0.76–1.89)
Prince Edward Island	0.50 (0.18–1.39)
Nova Scotia	0.70 (0.44–1.13)
New Brunswick	0.64 (0.41–1.02)
Ontario	Reference
Manitoba	0.32 (0.21–0.48)
Saskatchewan	0.60 (0.41–0.87)
Alberta	0.35 (0.27–0.46)
British Columbia	0.48 (0.37–0.62)
Territories	0.37 (0.11–1.23)

### Variability

Although the overall ICU admission rate was 3.2 cases per 1000 pregnancies, the range in ICU admission variability among all hospitals was wide—from 0 to 500 cases per 1000 pregnancies (Additional file 4: Table S4). There was a minimal variability in ICU admission rate at the provincial level (Additional file 5: Table S5). Using the median odds ratio, for 2 pregnant women with similar characteristics at different hospitals, the average (median) odds of being admitted to ICU was 1.92 in 1 hospital compared to another (Additional file 7: Table S7). The proportion of variability in ICU admission due to hospital-level factors, as denoted by the variance partitioning coefficient, was 33.3%, with substantial variation in the odds of ICU admission across hospitals (Additional file 7: Table S7). Furthermore, ICU admission rate for postpartum readmissions was relatively high (36.5 per 1000 pregnancies) in comparison with other periods of admissions, and ICU admission among abortion-related admissions had the highest variability (median odds ratio, 2.21) (Additional file 8: Table S8).

### Sensitivity analyses

All primary and secondary results were similar in sensitivity analyses, employing the number of pregnancy-related hospital admission in continuous variable (Additional file 9: Table S9), considering only the highest acuity ICUs (Additional file 10: Table S10), and using multiple imputation datasets to account for missing data (Additional file 11: Table S11).

## Discussion

In this study, we found that only 10% of the 15.8 women per 1000 pregnancies in Canada who experience a severe maternal morbidity event and only two thirds of the 6.2 women who die per 100,000 pregnancies are admitted to ICU. We also found wide hospital-level variability in ICU admission practices. Patients living in urban settings and of lower socioeconomic status were more likely to be admitted to ICU (Table 4).

**Table 4:** Box summary of key points

Question	
• Is there variability of ICU admission practices for pregnant and postpartum women in Canadian hospitals and what is the association between patient and hospital characteristics and ICU admission practices?	
Findings	
• In this nationwide population-based cohort study of 342 acute care hospitals with 3.1 million pregnancies in Canada, there were 3.2 ICU admissions per 1000 pregnancies with provincial variation. • More comorbidities, urban residence, and lower incomes were associated with ICU admission. • In comparison with the maternal comorbidity index = 0, increasing maternal comorbidity index has higher odds of admitting to ICU (index = 1, odds ratio 1.90; index = 2 or higher, odds ratio 8.67). • Median odds ratio of ICU admission among Canadian pregnant women was 1.92, which demonstrated for two pregnant women with similar characteristics at different hospitals; the median odds of being admitted to ICU in one hospital compared to another was 1.92. • Given the fact that only two thirds of all women who subsequently died were admitted to ICU, these happened to those who were less sick at the time of hospital admission and less likely to transfer to another institution for acute care, but more likely to develop a life-threatening condition in urban hospitals, compared to maternal death with ICU admission.	
Meaning	
• There is a substantial variability in admission patterns to intensive care for pregnant and postpartum women in Canadian hospitals.	

Discovering variability in clinical practice for pregnant and postpartum women is important as it presents an opportunity for improved quality of care. For example, identifying large variability in preoperative medical consultation has led to efforts to improve perioperative anesthetic care [38]. Systematic literature search has revealed that ours is the first population-based study to examine the variability of the care of critically ill pregnant and postpartum Canadian women and highlights modifiable factors in ICU admission practices (Table 5, Additional file 12: Table S12). Prior population-based studies of pregnancy-related ICU admissions in addition to ours show variability in ICU admission rates among identified countries, ranging from 3.2 to 39 per 1000 pregnancies (Table 5) [3, 39–42]. While greater maternal comorbidity was associated with increasing likelihood of ICU admission, a minority of patients with sepsis and eclampsia were admitted to ICU. It may be due to early recovery from a sick condition after the delivery in case delivery was the first step of management and/or different pathway of patient care for pregnant women. In other words, first being admitted to obstetrical general wards may delay an acute care. Although only two thirds of all women who subsequently died were admitted to ICU, some conditions of severe maternal morbidity (e.g., obstetric embolism) and subsequent death could occur very quickly and limit the potential for ICU admission (Additional file 6: Table S6), which were indicated by an additional analysis of maternal death without ICU admission (Additional file 13: Table S13). Maternal death without ICU admission happened to those who were less sick (i.e., less maternal comorbidity index) at the time of hospital admission and less likely to transfer to another institution for acute care, but more likely to develop a life-threatening condition (i.e., severe maternal morbidity) in urban hospitals, compared to maternal death with ICU admission. There were 15 maternal deaths in operating rooms without ICU admission, related to postpartum hemorrhage and obstetric embolism. On the other hand, our results found that other conditions that progress more slowly may inherently have a greater time period for clinicians to consider admission to ICU, and hence a greater potential for intervention and improved outcomes (e.g., sepsis) through quality improvements of implementation of sepsis bundles, for example [43]. These findings highlight a potential opportunity for earlier recognition by clinicians for women at risk and earlier recognition of the onset of critical illness. More practically, in comparison with MCI = 0, increasing maternal comorbidity index has higher odds of admitting to ICU (index = 1, odds ratio (OR) 1.90, 95%CI 1.79–2.02; index = 2 or higher, OR 8.67, 95%CI 8.24–9.12) (Additional file 14: Table S14). Our findings also have relevance to policymakers. Women living in rural and resource-limited locations may experience the greatest risk [2], highlighting additional opportunities for identification of those at risk, with potential solutions of a lower threshold for consultation, referral and increased frequency of follow-up care, outreach of tele-medicine [44, 45], ease of transportation for mothers to specialist care, and/or consideration of a mobile ICU to resource-limited hospitals [46].

**Table 5:** Research in context. Population-based studies of pregnancy-related ICU admissions

Studies	Study period	Study sites (country)	No. of pregnancy-related ICU patients	ICU admission rate	Mortality (per 100,000 cases)	Variability of ICU admission rate among hospitals
Harrison 2005	1995–2003	UK	1902	9.0 per 1000 ICU admissions	Hospital mortality (26 per 100,000 all ICU admissions, 3049 per 100,000 pregnancy-related ICU admissions)	Not described
Madan 2009	1997–2005	NJ, USA	15,447	15.4 per 1000 pregnancies	Hospital mortality (12 per 100,000 pregnancies, 149 per 100,000 pregnancy-related ICU admissions)	Not described
Wanderer 2013	1999–2008	MD, USA	2927	4.1 per 1000 pregnancies	Hospital mortality (7.6 per 100,000 pregnancies, 1810 per 100,000 pregnancy-related ICU admissions)	Not described
Chantry 2015	2006–2009	France	11,824	3.6 per 1000 pregnancies	ICU mortality (4.7 per 100,000 pregnancies, 1294 per 100,000 pregnancy-related ICU admissions)	Not described
Oud 2017	2001–2010	TX, USA	158,410	39 per 1000 pregnancies	Hospital mortality (10 per 100,000 pregnancies, 261 per 100,000 pregnancy-related ICU admissions)	Not described*
Aoyama 2019	2004–2015	Canada	10,204	3.2 per 1000 pregnancies	Hospital mortality (6.2 per 100,000 pregnancies, 1274 per 100,000 pregnancy-related ICU admissions)	MOR 1.92

Systematic search. We searched MEDLINE, EMBASE, and Cochrane databases for studies published until September 2019. Search terms for ICU, Pregnancy, Admission, and Big data were combined (detailed search strategy in Additional file 12: Table S12). The reference lists of the selected papers were hand-searched for other potentially relevant papers. We have summarized all the relevant studies here as well as in the main text, but we were mainly interested in population-based studies relevant to our research question

*Variability of pregnancy outcomes and categories of pregnancy-associated hospitalizations were reported

### Strengths and weaknesses of the study

Our study has several strengths. First, this is the first study based upon a nationwide cohort to explore hospital variability of pregnancy-related ICU admission using multi-level logistic regression models accounting for hospitals as clusters. Second, we adopted the perspective of an *episode of care*, to account for the entire pregnancy and postpartum course rather than one admission allowing use to determine outcomes from the entire pregnancy instead of outcomes relating to individual hospital admissions. Third, the maternal comorbidity index was well-validated risk adjustment tool in the DAD of CIHI, to account for known risk factors of maternal death and organ injury [24, 25, 47]. Fourth, maternal age younger than 20 years old and over 35 years old was associated with maternal mortality, and hence properly adjusted in our analyses [8]. Fifth, we employed the best risk adjustment tool for our dataset among existing risk prediction models including ICU admission scores [24, 48] and ensured a valid temporal relationship between the MCI and severe maternal morbidity/ICU admission. Last, we have used existing and well-validated health administrative datasets to describe pregnancies as well as ICU practice across diverse regions and over time [15–20].

This study also has limitations. First, certain variables that may be influential for maternal outcomes were unavailable in our dataset, for example, patient characteristics such as body mass index and pregnancy-associated factors such as the use of assisted reproductive technology, among others. However, obesity did not remain in the maternal comorbidity index to account for maternal death and organ injury during the original development [47]. Even among available variables, and considering patient and hospital factors, our predictive models for ICU admission do not explain all variability, highlighting the existence of other potentially important factors. We had a high rate of missing data for certain variables, particularly postal code markers of socioeconomic status. However, there were few differences among known characteristics between groups with missing and non-missing data, and our results did not differ when we used a multiply imputed dataset in our sensitivity analyses. CIHI did not provide structural characteristics for each of all 342 hospitals enrolled in this study such as detailed type of ICUs and existence of neonatal intensive care unit. And hence, groups according to the annual volume of their delivery admission were created, to characterize enrolled hospitals from an obstetric practice point of view. Additionally, ICU utilization and transfer rate in each group of hospitals were also calculated (Additional file 4: Table S4). It is possible that some diagnostic coding error exists, for example, over-coding of ARDS among patients managed in post-anesthesia care unit or labor and delivery room and not the ICU (only 76.2% of patients with ARDS were admitted to ICU). Canadian Coding Standards Evolution Chronicle reports that post-procedural respiratory disorder was used to be coded as ARDS [49]. Over-coding of ARDS may be attributed to this previous coding procedure. Probably, contemporary validation studies of ARDS by utilizing audits of hospital chart is required for future large epidemiological studies, since there was no such specific validation study of ARDS using CIHI DAD after the Berlin definition in 2012. Although the DAD does not contain specific information about the number of ICU beds in each acute care hospital, a previous study estimated the number of ICU beds per 100,000 population, by provinces, in 2009, ranging from 5.5 to 19.3 beds per 100,000 population [50]. Given the provincial ICU beds per 100,000 population in Canada, our study showed that there was a mostly linear relationship between provincial ICU capacity and maternal ICU admission rate (*r*^2^ = 0.74, *p* = 0.002) (Additional file 15: Figure S1), which implies that ICU capacity may influence the admission patterns of pregnancy-related ICU admission as well in Canada. Also, in this analysis, we did not explore neonatal outcomes—this will be an important next step. Finally, although CIHI collects data on each admission at all acute care hospitals across Canada, our findings may not generalize to the province of Quebec which maintains a unique database.

### Unanswered questions and future research

We found that only a minority of women who experience severe maternal morbidity are admitted to an intensive care unit in Canada, that there is a wide variability in ICU admission practice across hospitals and provinces, and that the greatest vulnerability exists for women at low socioeconomic status. These represent potentially modifiable factors for patients and should be the focus of quality of care improvement initiatives for clinicians and health systems.

## Conclusions

In conclusion of this study, we found that a minority of women who experience a severe maternal morbidity event and only two thirds of those who die are admitted to ICU. There is a wide hospital-level variability in ICU admission practices and that those living in urban locations and those of the lowest socioeconomic status were most likely to be admitted to ICU.

## Supplementary information

**Additional file 1:** **Table S1.** List of all variables used in this study.

**Additional file 2:** **Table S2.** The number of severe maternal morbidity events with ICU admission.

**Additional file 3:** **Table S3.** The number of severe maternal morbidity events with maternal death.

**Additional file 4:** **Table S4.** Characteristics of categorized 5 hospital groups according to hospital volume.

**Additional file 5:** **Table S5.** Characteristics of the cohort by province/territory.

**Additional file 6:** **Table S6.** Severe maternal morbidity diagnoses that could abruptly or slowly progress from clinical point of view.

**Additional file 7:** **Table S7.** Estimated regression coefficients and variance components for the multi-level mixed logistic regression models for the outcome of ICU admission [Outcome=ICU admission, main predictors= quintile of hospitals according to the number of pregnancy admission at each hospital].

**Additional file 8:** **Table S8.** Variability of ICU admission stratified by different type of admission.

**Additional file 9:** **Table S9.** Sensitivity analysis: Estimated regression coefficients and variance components for the multi-level mixed logistic regression models with the first modification of main predictors for the outcome of ICU admission [Outcome=ICU admission, main predictors=the number of pregnancy admission at each hospital (continuous variable)].

**Additional file 10:** **Table S10.** Sensitivity analysis: Estimated regression coefficients and variance components for the multi-level mixed logistic regression models with the second modification of main predictors for the outcome of highest acuity ICU admission [Outcome= highest acuity ICU admission, main predictors= quintile of hospitals according to the number of pregnancy admission at each hospital].

**Additional file 11:** **Table S11.** Sensitivity analysis: Estimated regression coefficients and variance components for the multi-level mixed logistic regression models for the outcome of ICU admission within multiple imputation datasets [Outcome=ICU admission, main predictors=quintile of hospitals according to the number of pregnancy admission at each hospital].

**Additional file 12:** **Table S12.** Search strategy in MEDLINE, EMBASE, Cochrane.

**Additional file 13:** **Table S13.** General characteristics of maternal death according to whether admitted to ICU or not.

**Additional file 14:** **Table S14.** Estimated odds ratios for multilevel logistic regression model for Intensive Care Unit admission with categorized Maternal Comorbidity Index.

**Additional file 15:** **Figure S1.** Provincial ICU Beds per 100,000 population and Provincial Intensive Care Unit Admissions per 1000 Live Births.

## Data Availability

All data and material can be accessed through the manuscript, supplemental information, and direct contact to the corresponding author, if needed.

## Abbreviations

CCI: Canadian Classification of Health Interventions
CI: Confidence intervals
CIHI: Canadian Institute of Health Information
DAD: Discharge Abstract Database
ICD-10CA: International Classification of Diseases and Related Health Problems Tenth Revision, Canada
ICU: Intensive care unit
IOR-80: Interval odds ratio-80
MCI: Maternal comorbidity index
MOR: Median odds ratio
OR: Odds ratio
VPC: Variance partitioning coefficient

## References

[1] Kassebaum NJ, Bertozzi-Villa A, Coggeshall MS, Shackelford KA, Steiner C, Heuton KR, Gonzalez-Medina D, Barber R, Huynh C, Dicker D, Templin T, Wolock TM, Ozgoren AA, Abd-Allah F, Abera SF, Abubakar I, Achoki T, Adelekan A, Ademi Z, Adou AK, Adsuar JC, Agardh EE, Akena D, Alasfoor D, Alemu ZA, Alfonso-Cristancho R, Alhabib S, Ali R, Al Kahbouri MJ, Alla F (2014). Global, regional, and national levels and causes of maternal mortality during 1990-2013: a systematic analysis for the global burden of disease study 2013. Lancet.

[2] Aoyama K, Ray JG, Pinto R, Hill A, Scales DC, Lapinsky SE, Fowler RA (2019). Temporal variations in incidence and outcomes of critical illness among pregnant and postpartum women in Canada: a population-based observational study. J Obstet Gynaecol Can.

[3] Wanderer JP, Leffert LR, Mhyre JM, Kuklina EV, Callaghan WM, Bateman BT (2013). Epidemiology of obstetric-related ICU admissions in Maryland: 1999-2008*. Crit Care Med.

[4] Pollock W, Rose L, Dennis C (2010). Pregnant and postpartum admissions to the intensive care unit: a systematic review. Intensive Care Med.

[5] Einav S, Bromiker R, Sela HY (2017). Maternal critical illness. Curr Anesthesiol Rep.

[6] Souza JP, Gülmezoglu AM, Vogel J, Carroli G, Lumbiganon P, Qureshi Z, Costa MJ, Fawole B, Mugerwa Y, Nafiou I, Neves I, Wolomby-Molondo JJ, Bang HT, Cheang K, Chuyun K, Jayaratne K, Jayathilaka CA, Mazhar SB, Mori R, Mustafa ML, Pathak LR, Perera D, Rathavy T, Recidoro Z, Roy M, Ruyan P, Shrestha N, Taneepanichsku S, Tien NV, Ganchimeg T (2013). Moving beyond essential interventions for reduction of maternal mortality (the WHO Multicountry Survey on Maternal and Newborn Health): a cross-sectional study. Lancet.

[7] Bateman BT (2019). What’s new in obstetric anesthesia: a focus on maternal morbidity and mortality. Int J Obstet Anesth.

[8] Aoyama K, Pinto R, Ray JG, Hill AD, Scales DC, Lapinsky SE, Hladunewich MA, Seaward GR, Fowler RA (2019). Association of maternal age with severe maternal morbidity and mortality in Canada. JAMA Netw Open.

[9] Gooch RA, Kahn JM (2014). ICU bed supply, utilization, and health care spending: an example of demand elasticity. JAMA.

[10] Rubenfeld GD, Rhodes A (2014). How many intensive care beds are enough?. Intensive Care Med.

[11] Tu JV, Bowen J, Chiu M, Ko DT, Austin PC, He Y, Hopkins R, Tarride J-E, Blackhouse G, Lazzam C, Cohen EA, Goeree R (2007). Effectiveness and safety of drug-eluting stents in Ontario. N Engl J Med.

[12] Nathens AB, Jurkovich GJ, Maier RV, Grossman DC, MacKenzie EJ, Moore M, Rivara FP (2001). Relationship between trauma center volume and outcomes. JAMA.

[13] Kahn JM, Goss CH, Heagerty PJ, Kramer AA, O’Brien CR, Rubenfeld GD (2006). Hospital volume and the outcomes of mechanical ventilation. N Engl J Med.

[14] Yarnell CJ, Fu L, Manuel D, Tanuseputro P, Stukel T, Pinto R, Scales DC, Laupacis A, Fowler RA (2017). Association between immigrant status and end-of-life care in Ontario, Canada. JAMA.

[15] Canadian Institute for Health Information (2012). Standards and data submission data quality documentation, Discharge Abstract Database—multi-year information.

[16] Canadian Institute for Health Information (2015). Data quality documentation, Discharge Abstract Database - current-year information, 2014–2015.

[17] Goel V, Williams J, Anderson G, Blackstien-Hirsch P, Fooks C, Naylor C (1996). Patterns of health care in Ontario.

[18] Scales DC, Thiruchelvam D, Kiss A, Redelmeier DA (2008). The effect of tracheostomy timing during critical illness on long-term survival. Crit Care Med.

[19] Juurlink D, Preyra C, Croxford R, Chong A, Austin P, Tu J, Laupacis A. Canadian Institute for Health Information Discharge Abstract Database: a validation study. Toronto Inst Clin Eval Sci. 2006:1–69.

[20] Urquia ML, Stukel TA, Fung K, Glazier RH, Ray JG (2011). Estimating gestational age at birth: a population-based derivation-validation study. Chronic Dis Inj Can.

[21] Garland A, Yogendran M, Olafson K, Scales DC, McGowan KL, Fransoo R (2012). The accuracy of administrative data for identifying the presence and timing of admission to intensive care units in a Canadian province. Med Care.

[22] Joseph K.S., Liu Shiliang, Rouleau Jocelyn, Kirby Russell S., Kramer Michael S., Sauve Reg, Fraser William D., Young David C., Liston Robert M. (2010). Severe Maternal Morbidity in Canada, 2003 to 2007: Surveillance Using Routine Hospitalization Data and ICD-10CA Codes. Journal of Obstetrics and Gynaecology Canada.

[23] Filippi V, Chou D, Barreix M, Say L (2018). A new conceptual framework for maternal morbidity. Int J Gynecol Obstet.

[24] Aoyama K, D’Souza R, Inada E, Lapinsky SE, Fowler RA (2017). Measurement properties of comorbidity indices in maternal health research: a systematic review. BMC Pregnancy Childbirth.

[25] Metcalfe A, Lix LM, Johnson JA, Currie G, Lyon AW, Bernier F, Tough SC (2015). Validation of an obstetric comorbidity index in an external population. BJOG An Int J Obstet Gynaecol.

[26] Yang D, Dalton J. A unified approach to measuring the effect size between two groups using SAS®. SAS Glob Forum. 2012;6:1–6.

[27] Austin PC, Merlo J (2017). Intermediate and advanced topics in multilevel logistic regression analysis. Stat Med.

[28] Austin PC, Goel V, Van Walraven C (2001). An introduction to multilevel regression models. Can J Public Heal.

[29] Larsen K, Merlo J (2005). Appropriate assessment of neighborhood effects on individual health: integrating random and fixed effects in multilevel logistic regression. Am J Epidemiol.

[30] Merlo J, Chaix B, Ohlsson H, Beckman A, Johnell K, Hjerpe P, Råstam L, Larsen K (2006). A brief conceptual tutorial of multilevel analysis in social epidemiology: using measures of clustering in multilevel logistic regression to investigate contextual phenomena. J Epidemiol Community Health.

[31] Vesin A, Azoulay E, Ruckly S, Vignoud L, Rusinovà K, Benoit D, Soares M, Azeivedo-Maia P, Abroug F, Benbenishty J, Timsit JF (2013). Reporting and handling missing values in clinical studies in intensive care units. Intensive Care Med.

[32] Haukoos JS, Newgard CD (2007). Advanced statistics: missing data in clinical research-part 1: an introduction and conceptual framework. Acad Emerg Med.

[33] Newgard CD, Haukoos JS (2007). Advanced statistics: missing data in clinical research-part 2: multiple imputation. Acad Emerg Med.

[34] Sterne J. A C, White I. R, Carlin J. B, Spratt M., Royston P., Kenward M. G, Wood A. M, Carpenter J. R (2009). Multiple imputation for missing data in epidemiological and clinical research: potential and pitfalls. BMJ.

[35] Lee KJ, Carlin JB (2010). Multiple imputation for missing data: fully conditional specification versus multivariate normal imputation. Am J Epidemiol.

[36] Graham JW, Olchowski AE, Gilreath TD (2007). How many imputations are really needed? Some practical clarifications of multiple imputation theory. Prev Sci.

[37] van Buuren S (2007). Multiple imputation of discrete and continuous data by fully conditional specification. Stat Methods Med Res.

[38] Wijeysundera Duminda N., Austin Peter C., Beattie W. Scott, Hux Janet E., Laupacis Andreas (2012). Variation in the Practice of Preoperative Medical Consultation for Major Elective Noncardiac Surgery. Anesthesiology.

[39] Harrison D, Penny J, Yentis S, Fayek S, Brady A (2005). Case mix, outcome and activity for obstetric admissions to adult, general critical care units: a secondary analysis of the ICNARC Case Mix Programme Database. Crit Care.

[40] Madan I, Puri I, Jain NJ, Grotegut C, Nelson D, Dandolu V (2009). Characteristics of obstetric intensive care unit admissions in New Jersey. J Matern Neonatal Med.

[41] Chantry AA, Deneux-Tharaux C, Bonnet MP, Bouvier-Colle MH (2015). Pregnancy-related ICU admissions in France: trends in rate and severity, 2006-2009. Crit Care Med.

[42] van Vliet EOG, Askie LA, Mol BWJ, Oudijk MA (2017). Antiplatelet agents and the prevention of spontaneous preterm birth: a systematic review and meta-analysis. Obstet Gynecol.

[43] Machado FR, Ferreira EM, Schippers P, de Paula IC, Saes LSV, de Oliveira FI, Tuma P, Nogueira Filho W, Piza F, Guare S, Mangini C, Guth GZ, Azevedo LCP, FGR F, JLG d A, Mansur NS, Salomão R. Implementation of sepsis bundles in public hospitals in Brazil: a prospective study with heterogeneous results. Crit Care. 2017;21(1):268. 10.1186/s13054-017-1858-z PMC5664817 29089025

[44] Wilcox M Elizabeth, Adhikari Neill KJ (2012). The effect of telemedicine in critically ill patients: systematic review and meta-analysis. Critical Care.

[45] Kovacevic P, Dragic S, Kovacevic T, Momcicevic D, Festic E, Kashyap R, Niven AS, Dong Y, Gajic O (2019). Impact of weekly case-based tele-education on quality of care in a limited resource medical intensive care unit. Crit Care.

[46] Wiegersma JS, Droogh JM, Zijlstra JG, Fokkema J, Ligtenberg JJM (2011). Quality of interhospital transport of the critically ill: impact of a mobile intensive care unit with a specialized retrieval team. Crit Care.

[47] Bateman BT, Mhyre JM, Hernandez-Diaz S, Huybrechts KF, Fischer MA, Creanga AA, Callaghan WM, Gagne JJ (2013). Development of a comorbidity index for use in obstetric patients. Obstet Gynecol.

[48] Aoyama K, D’Souza R, Pinto R, Ray JG, Hill A, Scales DC, Lapinsky SE, Seaward GR, Hladunewich M, Shah PS, Fowler RA (2018). Risk prediction models for maternal mortality: a systematic review and meta-analysis. PLoS One.

[49] Canadian Institute for Health Information: Canadian Coding Standards Evolution Chronicle. Ottawa; 2018

[50] Fowler RA, Abdelmalik P, Wood G, Foster D, Gibney N, Bandrauk N, Turgeon AF, Lamontagne F, Kumar A, Zarychanski R, Green R, Bagshaw SM, Stelfox HT, Foster R, Dodek P, Shaw S, Granton J, Lawless B, Hill A, Rose L, Adhikari NK, Scales DC, Cook DJ, Marshall JC, Martin C, Jouvet P, Canadian Critical Care Trials Group, Canadian ICU Capacity Group (2015). Critical care capacity in Canada: results of a national cross-sectional study. Crit Care.

