# Supplementary material for: Variability in intensive care unit admission among pregnant and postpartum women in Canada: a nationwide population-based observational study
Source: Crit Care. 2019 Nov 27;23:381. doi: 10.1186/s13054-019-2660-x (PMC6881971; doi:10.1186/s13054-019-2660-x)
Supplement: Supplementary file 1 — Additional file 1: Table S1. List of all variables used in this study. [file 13054_2019_2660_MOESM1_ESM.docx]

Table S1. List of all variables used in this study

| **Variable** | **Values** |
| --- | --- |
| **Patient-level variables** |  |
| Age in years | Continuous |
| Age categories | < 15 years, 15-19 years, 20-24 years, 25-29 years, 30-34 years, 35-39 years, 40-44 years, > 44 years |
| Maternal Comorbidity Index | Continuous (ranges from 0 to 45) |
| Parity categories | 0, 1, > 2 |
| Patient residence | 1 = Urban, 0 = Rural |
| Transfer status | 1 = Yes, 0 = No |
| Patient are income quintile | 1 (lowest), 2, 3, 4, 5 (highest) |
| **Hospital-level variables** |  |
| Hospital location | 1 = Urban, 0 = Rural |
| Province of Hospital location | Newfoundland and Labrador, Prince Edward Island, Nova Scotia, New Brunswick, Ontario, Manitoba, Saskatchewan, Alberta, British Columbia, Territories (Yukon, Northwest Territories and Nunavut) |
| Number of pregnancy related hospital admissions | Continuous |
| Hospital volume categories | 0 (less than 55 over the study period), 1 (lowest), 2, 3, 4, 5 (highest) |
| **Outcome variables** |  |
| Intensive care unit admission | 1 = Yes, 0 = No |
| Highest acuity Intensive care unit admission | 1 = Yes, 0 = No |
| Death | 1 = Yes, 0 = No |
| Severe maternal morbidity (any) | 1 = Yes, 0 = No |
| Severe maternal morbidity (count) | 0, 1, 2, 3, > 4 |
