# Supplementary material for: Variability in intensive care unit admission among pregnant and postpartum women in Canada: a nationwide population-based observational study
Source: Crit Care. 2019 Nov 27;23:381. doi: 10.1186/s13054-019-2660-x (PMC6881971; doi:10.1186/s13054-019-2660-x)
Supplement: Supplementary file 2 — Additional file 2: Table S2. The number of severe maternal morbidity events with ICU admission. [file 13054_2019_2660_MOESM2_ESM.docx]

Table S2. The number of severe maternal morbidity events with ICU admission

| **Admission type** | **No. of Severe maternal morbidity indicators** | **No. (%) with**  **ICU admission** |
| --- | --- | --- |
| **Any ICU admission** | 0 (N = 3,111,135) | 3,370 (0.1) |
|  | 1 (N = 44,319) | 2,334 (5.3) |
|  | 2 (N = 4,232) | 1,304 (30.8) |
|  | 3 (N = 1,342) | 716 (53.3) |
|  | > 4 (N = 1,275) | 927 (72.7) |
|  |  |  |
| **Highest acuity ICU admission** | 0 (N = 3,111,135) | 2,912 (0.1) |
|  | 1 (N = 44,319) | 2,102 (4.7) |
|  | 2 (N = 4,232) | 1,229 (29.0) |
|  | 3 (N = 1,342) | 691 (51.5) |
|  | > 4 (N = 1,275) | 901 (70.7) |

ICU: Intensive Care Unit
