# Supplementary material for: Variability in intensive care unit admission among pregnant and postpartum women in Canada: a nationwide population-based observational study
Source: Crit Care. 2019 Nov 27;23:381. doi: 10.1186/s13054-019-2660-x (PMC6881971; doi:10.1186/s13054-019-2660-x)
Supplement: Supplementary file 3 — Additional file 3: Table S3. The number of severe maternal morbidity events with maternal death. [file 13054_2019_2660_MOESM3_ESM.docx]

Table S3. The number of severe maternal morbidity events with maternal death

| **Hospital course** | **No. of Severe maternal morbidity indicators** | **No. (%) of deaths** |
| --- | --- | --- |
| **Any ICU admission** | 0 (N = 3,111,135) | 1 (0.03) |
|  | 1 (N = 44,319) | 14 (0.60) |
|  | 2 (N = 4,232) | 29 (2.22) |
|  | 3 (N = 1,342) | 25 (3.49) |
|  | > 4 (N = 1,275) | 61 (6.58) |
|  |  |  |
| **No ICU admission** | 0 (N = 3,111,135) | 13 (0) |
|  | 1 (N = 44,319) | 20 (0.05) |
|  | 2 (N = 4,232) | 10 (0.34) |
|  | 3 (N = 1,342) | 6 (0.96) |
|  | > 4 (N = 1,275) | 16 (4.60) |

ICU: Intensive Care Unit
