# Supplementary material for: Variability in intensive care unit admission among pregnant and postpartum women in Canada: a nationwide population-based observational study
Source: Crit Care. 2019 Nov 27;23:381. doi: 10.1186/s13054-019-2660-x (PMC6881971; doi:10.1186/s13054-019-2660-x)
Supplement: Supplementary file 4 — Additional file 4: Table S4. Characteristics of categorized 5 hospital groups according to hospital volume. [file 13054_2019_2660_MOESM4_ESM.docx]

Table S4-1. Characteristics of hospital groups according to increasing (1->5) hospital volume

|  | Hospital quintile | | | | | |
| --- | --- | --- | --- | --- | --- | --- |
|  | Group 1 | Group 2 | Group 3 | Group 4 | Group 5 | Standardized difference† |
| The number of Hospitals | 99 | 38 | 68 | 68 | 69 | N/A |
| The number of patients at each hospital over 11 years (median, 25thm 75^th^ percentile) | 183 (107, 265) | 975 (788, 1,386) | 3,221 (2,461, 3,696) | 9,512 (7,079, 12,462) | 35,611 (26,144, 57,951) | N/A |
| Intensive care unit admission (count, %) | 141 (1.0) | 203 (0.3) | 818 (0.4) | 2,165 (0.4) | 6,814 (0.3) | - 0.08 |
| Severe Maternal Morbidity (count, %) | 292 (2.0) | 1,117 (1.7) | 3,591 (1.7) | 8,981 (1.5) | 35,908 (1.6) | - 0.02 |
| Death (count, %) | 6 (0.04) | 5 (0.01) | 11 (0.01) | 29 (0.00) | 142 (0.01) | - 0.02 |
|  |  |  |  |  |  |  |
| Patient variables |  |  |  |  |  |  |
| Age, years (mean, SD) | 27.6 (6.1) | 27.3 (5.5) | 27.5 (5.6) | 28.3 (5.6) | 30.1 (5.5) | - 0.06 |
| Maternal comorbidity index (mean, SD) | 0.29 (0.71) | 0.29 (0.61) | 0.34 (0.69) | 0.39 (0.75) | 0.51 (0.89) | - 0.01 |
| Parity (count, %) |  |  |  |  |  | 0.14 |
| 0 | 8,229 (56.2) | 34,409 (51.3) | 130,973 (61.7) | 380,139 (63.2) | 1,279,009 (56.3) |  |
| 1 | 2,802 (19.2) | 16,670 (24.9) | 44,622 (21.0) | 128,955 (21.5) | 626,239 (27.7) |  |
| >=2 | 3,594 (24.6) | 15,976 (23.8) | 36,615 (23.8) | 91,920 (15.3) | 357,098 (15.8) |  |
| Residence (Urban) (count, %) | 6,833 (46.9) | 33,631 (50.3) | 110,312 (52.2) | 407,827 (68.3) | 2,007,185 (89.5) | 0.07 |
| Transfer (count, %) | 2,096 (14.3) | 4,859 (7.2) | 7,689 (3.6) | 10,447 (1.7) | 13,456 (0.6) | - 0.23 |
| Income quintile (count, %) |  |  |  |  |  | 0.24 |
| 1 (lowest) | 4,474 (32.4) | 14,385 (23.0) | 48,956 (24.8) | 120,148 (22.8) | 498,047 (26.0) |  |
| 2 | 2,130 (15.4) | 11,337 (18.1) | 38,250 (19.4) | 114,617 (21.7) | 406,474 (21.2) |  |
| 3 | 2,187 (15.8) | 12,690 (20.3) | 37,000 (18.7) | 107,028 (20.3) | 365,325 (19.1) |  |
| 4 | 2,652 (19.2) | 11,052 (17.6) | 35,819 (18.2) | 100,731 (19.1) | 343,848 (18.0) |  |
| 5 (highest) | 2,354 (17.1) | 13,139 (21.0) | 37,260 (18.9) | 84,557 (16.0) | 298,944 (15.6) |  |
|  |  |  |  |  |  |  |
| Hospital variables |  |  |  |  |  |  |
| Province** |  |  |  |  |  | 0.56 |
| Hospital (Urban) (count, %) | 10,235 (70.0) | 50,791 (75.7) | 188,403 (88.8) | 581,264 (96.7) | 2,242,956 (100.0) | 0.13 |

*: Data are presented as mean (SD), median [25^th^, 75^th^ percentile] or count (%).

**: See Appendix D-2

†: Standardized difference = difference in means or proportions divided by standard error. Standardized mean differences of 0.2, 0.5, and 0.8 are often/generally equated to effect sizes of small, medium, and large

Table S4-2. Characteristics of hospital groups according to according to increasing (1->5) hospital volume

|  | Hospital quintile | | | | | |
| --- | --- | --- | --- | --- | --- | --- |
|  | Group 1 | Group 2 | Group 3 | Group 4 | Group 5 |  |
| Hospital variables |  |  |  |  |  |  |
| Province (count, %) |  |  |  |  |  | Total* |
| Newfoundland and Labrador | 385 (0.7) | 3,714 (7.2) | 13,994 (27.2) | 11,826 (23.0) | 21,549 (41.9) | 51,468 (100) |
| Prince Edward Island | 0 | 0 | 0 | 15,421 (100) | 0 | 15,421 (100) |
| Nova Scotia | 272 (0.3) | 0 | 14,446 (15.1) | 29, 726 (31.0) | 51,336 (53.6) | 95,780 (100) |
| New Brunswick | 372 (0.5) | 1,610 (2.0) | 9,271 (11.5) | 33,223 (41.3) | 35,920 (44.7) | 80,396 (100) |
| Ontario | 2,916 (0.2) | 15,113 (1.0) | 66,198 (4.3) | 251,626 (16.4) | 1,193,384 (78.0) | 1,529,237 (100) |
| Manitoba | 1,277 (0.7) | 3,811 (2.1) | 16,641 (9.2) | 40,508 (22.4) | 118,237 (65.5) | 180,474 (100) |
| Saskatchewan | 2,452 (1.5) | 6,170 (3.9) | 11,532 (7.3) | 29,425 (18.6) | 108,540 (68.6) | 158,119 (100) |
| Alberta | 3,374 (0.6) | 27,419 (4.9) | 39,736 (7.2) | 41,691 (7.5) | 442,034 (79.7) | 554,254 (100) |
| British Columbia | 3,327 (0.7) | 7,737 (1.6) | 40,392 (8.5) | 131,217 (27.7) | 291,344 (61.5) | 474,017 (100) |
| Territories | 250 (1.4) | 1,481 (8.2) | 0 | 16,351 (90.4) | 0 | 18,082 (100) |

*: proportion of each cell sums up to 100% in each row (i.e. each province)
