# Supplementary material for: Variability in intensive care unit admission among pregnant and postpartum women in Canada: a nationwide population-based observational study
Source: Crit Care. 2019 Nov 27;23:381. doi: 10.1186/s13054-019-2660-x (PMC6881971; doi:10.1186/s13054-019-2660-x)
Supplement: Supplementary file 5 — Additional file 5: Table S5. Characteristics of the cohort by province/territory. [file 13054_2019_2660_MOESM5_ESM.docx]

Table S5. Characteristics of the cohort by province/territory

|  | Province | | | | | | | | | | Total |
| --- | --- | --- | --- | --- | --- | --- | --- | --- | --- | --- | --- |
|  | Newfoundland and Labrador | Prince Edward Island | Nova Scotia | New Brunswick | Ontario | Manitoba | Saskatchewan | Alberta | British Columbia | Territories |  |
| The number of pregnancy episodes | 51,468 | 15,421 | 95,780 | 80,396 | 1,529,237 | 180,474 | 158,119 | 554,254 | 474,017 | 18.082 | 3,157,248 |
| Intensive care unit admissions (count, %) | 256 (0.5) | 39 (0.2) | 223 (0.2) | 297 (0.4) | 5,936 (0.4) | 371 (0.2) | 484 (0.3) | 1,176 (0.2) | 1,301 (0.3) | 59 (0.3) | 10,141 |
| Severe Maternal Morbidity (count, %) | 955 (1.9) | 252 (1.6) | 1,456 (1.5) | 1,217 (1.5) | 22,347 (1.5) | 3,096 (1.7) | 2,849 (1.8) | 10,081 (1.8) | 7,240 (1.5) | 396 (2.2) | 49,889 |
| Death (count, %) | 5 (0.01) | 0 (0.00) | 5 (0.01) | 2 (0.00) | 106 (0.01) | 9 (0.00) | 11 (0.01) | 30 (0.01) | 24 (0.01) | 1 (0.01) | 193 |
|  |  |  |  |  |  |  |  |  |  |  |  |
| Patient variables |  |  |  |  |  |  |  |  |  |  |  |
| Age, years (mean, SD) | 28.5 (5.6) | 28.6 (5.5) | 28.7 (5.7) | 28.0 (5.5) | 30.1 (5.5) | 27.9 (5.9) | 27.5 (5.7) | 29.0 (5.5) | 30.2 (5.6) | 27.0 (6.3) | 29.5 (5.6) |
| Maternal comorbidity  index (mean, SD) | 0.42 (0.78) | 0.42 (0.76) | 0.44 (0.80) | 0.37 (0.73) | 0.51 (0.87) | 0.38 (0.76) | 0.36 (0.76) | 0.45 (0.83) | 0.53 (0.87) | 0.32 (0.70) | 0.47(0.84) |
| Parity (count, %) |  |  |  |  |  |  |  |  |  |  |  |
| 0 | 50,483 (98.1) | 14,621 (94.8) | 92,342 (96.4) | 77,301 (96.1) | 717,840 (46.9) | 77,350 (42.9) | 67,769 (42.9) | 260,582 (47.0) | 466,389 (98.4) | 8,082 (44.7) | 1,832,759 (58.0) |
| 1 | 700 (1.4) | 494 (3.2) | 2,187 (2.3) | 2,193 (2.7) | 526,311 (34.4) | 52,244 (28.9) | 48,334 (30.6) | 177,575 (32.0) | 4,561 (1.0) | 4,689 (25.9) | 819,288 (26.0) |
| >=2 | 285 (0.5) | 306 (2.0) | 1,251 (1.3) | 902 (1.1) | 285,086 (18.6) | 50.880 (28.2) | 42,016 (26.6) | 116,097 (21.0) | 3,067 (0.7) | 5,311 (29.4) | 505,201 (16.0) |
| Residence (Urban)  (count, %) | 32,245 (63.1) | 5,987 (38.9) | 57,094 (60.0) | 43,917 (55.0) | 1,327,620 (87.5) | 118,193 (65.7) | 108,295 (68.7) | 452,355 (82.3) | 412,075 (87.7) | 8,007 (44.4) | 2,565,788 (81.9) |
| Transfer (count, %) | 615 (1.2) | 268 (1.7) | 1,255 (1.3) | 905 (1.1) | 10,720 (0.7) | 3,352 (1.9) | 2,911 (1.8) | 8,570 (1.5) | 9,492 (2.0) | 459 (2.5) | 38,547 (1.2) |
| Income quintile  (count, %) |  |  |  |  |  |  |  |  |  |  |  |
| 1 (lowest) | 8,771 (17.9) | 2,332 (15.2) | 18,651 (23.8) | 17,345 (22.4) | 322,946 (24.9) | 52,112 (30.4) | 43,875 (29.2) | 12,104 (27.4) | 97,668 (22.8) | 1,206 (37.0) | 686,010 (25.3) |
| 2 | 11,198 (22.8) | 2,964 (19.3) | 15,497 (19.8) | 15,226 (19.7) | 271,467 (20.9) | 32,768 (19.1) | 30,878 (20.6) | 96,976 (22.0) | 95,532 (22.3) | 302 (9.3) | 572,808 (21.1) |
| 3 | 8,494 (17.3) | 4,029 (26.2) | 14,556 (18.6) | 15,060 (19.4) | 253,376 (19.5) | 33,431 (19.5) | 26,490 (17.6) | 81,560 (18.5) | 86,273 (20.1) | 960 (29.4) | 524,228 (19.3) |
| 4 | 9,674 (19.7) | 2,399 (15.6) | 15,327 (19.6) | 14,945 (19.3) | 240,176 (18.5) | 29,544 (17.2) | 23,567 (15.7) | 77,079 (17.5) | 80,869 (18.9) | 522 (16.0) | 494,102 (18.2) |
| 5 (highest) | 10,887 (22.2) | 3,633 (23.7) | 14,337 (18.3) | 14,834 (19.2) | 210,547 (16.2) | 23,743 (13.8) | 25,238 (16.8) | 64,440 (14.6) | 68,325 (15.9) | 270 (8.3) | 436,254 (16.1) |
|  |  |  |  |  |  |  |  |  |  |  |  |
| Hospital variables |  |  |  |  |  |  |  |  |  |  |  |
| Hospital (Urban)  (count, %) | 47,443 (92.2) | 15,421 (100) | 93,200 (97.3) | 78,240 (97.3) | 1,507,758 (98.6) | 179,614 (99.5) | 155,665 (98.4) | 533,903 (96.3) | 448,876 (98.7) | 13,529 (74.8) | 3,073,649 (97.9) |
| Hospital group  according to hospital  volume of pregnancy |  |  |  |  |  |  |  |  |  |  |  |
| 1 (lowest) | 385 (0.7) | 0 | 272 (0.3) | 372 (0.5) | 2,916 (0.2) | 1,277 (0.7) | 2,452 (1.5) | 3,374 (0.6) | 3,327 (0.7) | 250 (1.4) | 14,625 |
| 2 | 3,714 (7.2) | 0 | 0 | 1,610 (2.0) | 15,113 (1.0) | 3,811 (2.1) | 6,170 (3.9) | 27,419 (4.9) | 7,737 (1.6) | 1,481 (8.2) | 67,055 |
| 3 | 13,994 (27.2) | 0 | 14,446 (15.1) | 9,271 (11.5) | 66,198 (4.3) | 16,641 (9.2) | 11,532 (7.3) | 39,736 (7.2) | 40,392 (8.5) | 0 | 212,210 |
| 4 | 11,826 (23.0) | 15,421 (100) | 29, 726 (31.0) | 33,223 (41.3) | 251,626 (16.4) | 40,508 (22.4) | 29,425 (18.6) | 41,691 (7.5) | 131,217 (27.7) | 16,351 (90.4) | 601,014 |
| 5 (highest) | 21,549 (41.9) | 0 | 51,336 (53.6) | 35,920 (44.7) | 1,193,384 (78.0) | 118,237 (65.5) | 108,540 (68.6) | 442,034 (79.7) | 291,344 (61.5) | 0 | 2,262,344 |

Data are presented as mean (SD), median [25^th^, 75^th^ percentile] or count (%).
