# Supplementary material for: Variability in intensive care unit admission among pregnant and postpartum women in Canada: a nationwide population-based observational study
Source: Crit Care. 2019 Nov 27;23:381. doi: 10.1186/s13054-019-2660-x (PMC6881971; doi:10.1186/s13054-019-2660-x)
Supplement: Supplementary file 6 — Additional file 6: Table S6. Severe maternal morbidity diagnoses that could abruptly or slowly progress from clinical point of view. [file 13054_2019_2660_MOESM6_ESM.docx]

Table S6. Severe maternal morbidity diagnoses that could abruptly or slowly progress from clinical point of view*

(a) Severe maternal morbidity diagnoses that could abruptly progress

| **Individual indicator of several maternal morbidity** | **No. (%) of patients with the individual Severe maternal morbidity indicator among the 50,012 severe maternal morbidity events** | **No. (%) of deaths among patients with the individual severe maternal morbidity indicator** | **No. (%) of deaths without ICU admission among patients dying with the individual severe maternal morbidity indicator** |
| --- | --- | --- | --- |
| Postpartum hemorrhage and hysterectomy | 1,561 (0.05) | 16 (1.02) | 8 (50.0) |
| Obstetric embolism | 1,404 (0.04) | 34 (2.42) | 16 (47.0) |
| Myocardial infarction, failure, arrest or pulmonary edema | 4,483 (0.14) | 101 (2.25) | 33 (32.7) |
| Obstetric shock | 1,124 (0.04) | 38 (3.38) | 6 (15.8) |
| Cerebrovascular diseases: subarachnoid and intracranial hemorrhage, cerebral infarction, stroke | 331 (0.01) | 30 (9.06) | 3 (10.0) |

(b) Severe maternal morbidity diagnoses that could slowly progress

| **Individual indicator of several maternal morbidity** | **No. (%) of patients with the individual Severe maternal morbidity indicator among the 50,012 severe maternal morbidity events** | **No. (%) of deaths among patients with the individual severe maternal morbidity indicator** | **No. (%) of deaths without ICU admission among patients dying with the individual severe maternal morbidity indicator** |
| --- | --- | --- | --- |
| Sepsis | 11,557 (0.37) | 21 (0.18) | 3 (14.3) |
| Cardiomyopathy in the puerperium | 1,058 (0.03) | 9 (0.85) | 1 (11.1) |
| Hepatic failure | 114 (0.00) | 14 (12.28) | 1 (7.1) |
| Acute Renal Failure | 1,116 (0.04) | 45 (4.03) | 1 (2.2) |
| Status asthmaticus | 76 (0.00) | 1 (1.32) | 0 (0) |

*Categories are not mutually exclusive
