# Supplementary material for: Variability in intensive care unit admission among pregnant and postpartum women in Canada: a nationwide population-based observational study
Source: Crit Care. 2019 Nov 27;23:381. doi: 10.1186/s13054-019-2660-x (PMC6881971; doi:10.1186/s13054-019-2660-x)
Supplement: Supplementary file 7 — Additional file 7: Table S7. Estimated regression coefficients and variance components for the multi-level mixed logistic regression models for the outcome of ICU admission [Outcome=ICU admission, main predictors= quintile of hospitals according to the number of pregnancy admission at each hospital]. [file 13054_2019_2660_MOESM7_ESM.docx]

Table S7. Estimated regression coefficients and variance components for the multi-level mixed logistic regression models for the

outcome of Intensive care unit (ICU) admission [Outcome=ICU admission, main predictors= quintile of hospitals according to the number of pregnancy admission at each hospital]

| Variable | Model 1 | Model 2 for ICU admission | | Model 3 for ICU admission with Hospital group according to Hospital pregnancy volume | |
| --- | --- | --- | --- | --- | --- |
|  | (Outcome=ICU admission) | Regression coefficient (95% CI) | P-value | Regression coefficient (95% CI) | P-value |
| Intercept | - 5.84 (- 5.95, - 5.73) | - 6.44(- 6.58, - 6.31) | <0.0001 | - 6.51 (- 6.91, - 6.12) | <0.0001 |
| Patient variables |  | | | | |
| Maternal Comorbidity Index |  | 0.63 (0.62, 0.64) | <0.0001 | 0.63 (0.62, 0.64) | <0.0001 |
| Age, mean years |  |  |  |  |  |
| < 15 |  | 0.54 (-0.27, 1.36) | 0.1918 | 0.55 (- 0.27, 1.36) | 0.1902 |
| 15-19 |  | 0.11 (0.00, 0.22) | 0.0591 | 0.11 (0.00, 0.22) | 0.0589 |
| 20-24 |  | Reference |  | Reference |  |
| 25-29 |  | 0.04 (- 0.03, 0.12) | 0.2147 | 0.04 (- 0.03, 0.11) | 0.2672 |
| 30-34 |  | 0.18 (0.11, 0.25) | <0.0001 | 0.17 (0.10, 0.25) | <0.0001 |
| 35-39 |  | 0.40 (0.32, 0.48) | <0.0001 | 0.40 (0.32, 0.48) | <0.0001 |
| 40-44 |  | 0.79 (0.69, 0.90) | <0.0001 | 0.80 (0.69, 0.91) | <0.0001 |
| > 44 |  | 1.06 (0.80, 1.32) | <0.0001 | 1.07 (0.80, 1.33) | <0.0001 |
| Parity |  | - 0.24 (- 0.28, - 0.21) | <0.0001 | - 0.24 (- 0.28, - 0.21) | <0.0001 |
| Residence (urban versus rural) |  | 0.09 (0.02, 0.16) | 0.0083 | 0.08 (0.02, 0.15) | 0.0144 |
| Transfer |  | 2.54 (2.48, 2.61) | <0.0001 | 2.56 (2.49, 2.63) | <0.0001 |
| Income quintile |  |  |  |  |  |
| 1 (lowest) |  | 0.36 (0.29, 0.43) | <0.0001 | 0.37 (0.29, 0.44) | <0.0001 |
| 2 |  | 0.25 (0.18, 0.33) | <0.0001 | 0.25 (0.18, 0.33) | <0.0001 |
| 3 |  | 0.17 (0.10, 0.25) | <0.0001 | 0.18 (0.10, 0.25) | <0.0001 |
| 4 |  | 0.11 (0.03, 0.19) | 0.0064 | 0.11 (0.03, 0.19) | 0.0059 |
| 5 (highest) |  | Reference |  | Reference |  |
| Hospital variables |  | | | | |
| Groups according to Hospital volume of pregnancy |  |  |  |  |  |
| 1 (lowest volume) |  |  |  | 0.74 (0.37, 1.12) | 0.0001 |
| 2 |  |  |  | Reference |  |
| 3 |  |  |  | 0.31 (0.00, 0.62) | 0.0511 |
| 4 |  |  |  | 0.41 (0.10, 0.73) | 0.0108 |
| 5 (highest volume) |  |  |  | 0.27 (- 0.04, 0.59) | 0.0847 |
| Province |  |  |  |  |  |
| Newfoundland and Labrador |  |  |  | 0.18 (- 0.27, 0.64) | 0.4284 |
| Prince Edward Island |  |  |  | - 0.70 (- 1.73, 0.33) | 0.1811 |
| Nova Scotia |  |  |  | - 0.35 (- 0.83, 0.13) | 0.1480 |
| New Brunswick |  |  |  | - 0.44 (- 0.90, 0.02) | 0.0619 |
| Ontario |  |  |  | Reference |  |
| Manitoba |  |  |  | - 1.15 (- 1.57, - 0.73) | <0.0001 |
| Saskatchewan |  |  |  | - 0.51 (- 0.88, - 0.14) | 0.0066 |
| Alberta |  |  |  | - 1.05 (- 1.32, - 0.78) | <0.0001 |
| British Columbia |  |  |  | - 0.73 (- 0.98, - 0.47) | <0.0001 |
| Territories |  |  |  | - 1.00 (- 2.20, 0.21) | 0.1050 |
| Hospital (Urban versus rural) |  |  |  | 0.26 (- 0.06, 0.58) | 0.1089 |
|  |  |  |  |  |  |
| Variance of random effects | 0.7504 | 0.7253 | | 0.4658 | |
| Variance partition coefficient | 0.1857 | 0.1862 | | 0.1240 | |
| Median odds ratio | 2.29 | 2.29 | | 1.92 | |
| Interval odds ratio-80 for the groups |  |  | |  | |
| 1 (lowest volume) |  |  | | 0.61 – 7.23 | |
| 2 |  |  | | Reference | |
| 3 |  |  | | 0.39 – 4.70 | |
| 4 |  |  | | 0.44 – 5.19 | |
| 5 (highest volume) |  |  | | 0.38 – 4.53 | |

VPC: variance partition coefficient = Intra-class Correlation Coefficient, MOR: median odds ratio; ICU: intensive care unit
