# Supplementary material for: Variability in intensive care unit admission among pregnant and postpartum women in Canada: a nationwide population-based observational study
Source: Crit Care. 2019 Nov 27;23:381. doi: 10.1186/s13054-019-2660-x (PMC6881971; doi:10.1186/s13054-019-2660-x)
Supplement: Supplementary file 8 — Additional file 8: Table S8. Variability of ICU admission stratified by different type of admission. [file 13054_2019_2660_MOESM8_ESM.docx]

Table S8. Variability of ICU admission stratified by different type of admission, derived from the final multi-level mixed logistic regression models for the outcome of ICU admission [Outcome=ICU admission, predictors=same as the model 3 of primary analysis in Table S6]

|  | Type of admission | | | | |
| --- | --- | --- | --- | --- | --- |
|  | All | Antepartum* | Delivery*† | Postpartum*†† | Abortion* |
| The number of pregnancy episodes | 3,157,248 | 250,384 | 3,026,128 | 60,641 | 106,660 |
| The number of ICU admissions | 10,141 | 2,210 | 7,707 | 2,214 | 775 |
| ICU admission rate per 1,000 pregnancies | 3.2 | 8.8 | 2.5 | 36.5 | 7.3 |
| Variance of random effects | 0.4658 | 0.2700 | 0.3459 | 0.2716 | 0.6898 |
| Variance partition coefficients | 0.124 | 0.075 | 0.095 | 0.076 | 0.173 |
| Median odds ratio | 1.92 | 1.64 | 1.75 | 1.64 | 2.21 |

ICU: Intensive Care Unit

*Categories are not mutually exclusive

†: delivery or peripartum period

††: postpartum readmission
