# Supplementary material for: Variability in intensive care unit admission among pregnant and postpartum women in Canada: a nationwide population-based observational study
Source: Crit Care. 2019 Nov 27;23:381. doi: 10.1186/s13054-019-2660-x (PMC6881971; doi:10.1186/s13054-019-2660-x)
Supplement: Supplementary file 9 — Additional file 9: Table S9. Sensitivity analysis: Estimated regression coefficients and variance components for the multi-level mixed logistic regression models with the first modification of main predictors for the outcome of ICU admission [Outcome=ICU admission, main predictors=the number of pregnancy admission at each hospital (continuous variable)]. [file 13054_2019_2660_MOESM9_ESM.docx]

Table S9. Sensitivity analysis (the number of pregnancy admissions at each hospital in continuous format as a predictor of ICU admission): Estimated regression coefficients and variance components for the multi-level mixed logistic regression models with different first modified predictors for the outcome of Intensive care unit (ICU) admission [Outcome=ICU admission, main predictors=the number of pregnancy admission at each hospital (continuous variable)]

| Variable | Model 3 for ICU admission with Hospital group according to Hospital pregnancy volume | |
| --- | --- | --- |
|  | Regression coefficient (95% CI) | P-value |
| Intercept | - 6.18 (- 6.53, - 5.83) | <0.0001 |
| Patient variables |  | |
| Maternal Comorbidity Index | 0.63 (0.62, 0.64) | <0.0001 |
| Age, mean years |  |  |
| < 15 | 0.54 (- 0.27, 1.36) | 0.1917 |
| 15-19 | 0.11 (0.00, 0.22) | 0.0590 |
| 20-24 | Reference |  |
| 25-29 | 0.04 (- 0.03, 0.11) | 0.2700 |
| 30-34 | 0.18 (0.10, 0.25) | <0.0001 |
| 35-39 | 0.40 (0.32, 0.48) | <0.0001 |
| 40-44 | 0.80 (0.69, 0.91) | <0.0001 |
| > 44 | 1.07 (0.81, 1.33) | <0.0001 |
| Parity | - 0.24 (- 0.28, - 0.21) | <0.0001 |
| Residence (urban versus rural) | 0.08 (0.02, 0.15) | 0.0142 |
| Transfer | 2.56 (2.49, 2.63) | <0.0001 |
| Income quintile |  |  |
| 1 (lowest) | 0.34 (0.27, 0.42) | <0.0001 |
| 2 | 0.26 (0.18, 0.34) | <0.0001 |
| 3 | 0.17 (0.09, 0.26) | <0.0001 |
| 4 | 0.11 (0.03, 0.19) | 0.0092 |
| 5 (highest) | Reference |  |
| Hospital variables |  | |
| The number of pregnancy admissions at each hospital | -1.38E-6 | <0.0001 |
| Province |  |  |
| Newfoundland and Labrador | 0.12 (- 0.35, 0.59) | 0.6212 |
| Prince Edward Island | - 0.62 (- 1.67, 0.44) | 0.2517 |
| Nova Scotia | - 0.28 (- 0.77, 0.21) | 0.2671 |
| New Brunswick | - 0.43 (- 0.91, 0.05) | 0.0807 |
| Ontario | Reference |  |
| Manitoba | - 1.14 (- 1.58, - 0.71) | <0.0001 |
| Saskatchewan | - 0.48 (- 0.86, - 0.10) | 0.0127 |
| Alberta | - 1.10 (- 1.38, - 0.83) | <0.0001 |
| British Columbia | - 0.72 (- 0.98, - 0.46) | <0.0001 |
| Territories | - 0.91 (- 2.14, 0.32) | 0.1488 |
| Hospital (Urban versus rural) | 0.26 (- 0.06, 0.59) | 0.1103 |
| Variance of random effects | 0.5104 | |
| Variance partition coefficient | 0.13430 | |
| Median odds ratio | 1.98 | |
