# Supplementary material for: Variability in intensive care unit admission among pregnant and postpartum women in Canada: a nationwide population-based observational study
Source: Crit Care. 2019 Nov 27;23:381. doi: 10.1186/s13054-019-2660-x (PMC6881971; doi:10.1186/s13054-019-2660-x)
Supplement: Supplementary file 10 — Additional file 10: Table S10. Sensitivity analysis: Estimated regression coefficients and variance components for the multi-level mixed logistic regression models with the second modification of main predictors for the outcome of highest acuity ICU admission [Outcome= highest acuity ICU admission, main predictors= quintile of hospitals according to the number of pregnancy admission at each hospital]. [file 13054_2019_2660_MOESM10_ESM.docx]

Table S10. Sensitivity analysis: Estimated regression coefficients and variance components for the multi-level mixed logistic regression models with second modified main predictors for the outcome of highest acuity Intensive care unit (ICU) admission [Outcome= highest acuity ICU admission, main predictors= quintile of hospitals according to the number of pregnancy admission at each hospital]

| Variable | Model 3 for ICU admission with Hospital group according to Hospital pregnancy volume | |
| --- | --- | --- |
|  | Regression coefficient (95% CI) | P-value |
| Intercept | - 6.58 (- 6.97, - 6.19) | <0.0001 |
| Patient variables |  | |
| Maternal Comorbidity Index | 0.63 (0.61, 0.64) | <0.0001 |
| Age, mean years |  |  |
| < 15 | 0.63 (- 0.19, 1.45) | 0.1325 |
| 15-19 | 0.14 (0.02, 0.25) | 0.0209 |
| 20-24 | Reference |  |
| 25-29 | 0.03 (- 0.04, 0.11) | 0.3497 |
| 30-34 | 0.17 (0.09, 0.24) | <0.0001 |
| 35-39 | 0.39 (0.31, 0.47) | <0.0001 |
| 40-44 | 0.78 (0.67, 0.90) | <0.0001 |
| > 44 | 0.95 (0.66, 1.23) | <0.0001 |
| Parity | - 0.23 (- 0.26, - 0.19) | <0.0001 |
| Residence (urban versus rural) | 0.08 (0.01, 0.15) | 0.0247 |
| Transfer | 2.55 (2.48, 2.62) | <0.0001 |
| Income quintile |  |  |
| 1 (lowest) | 0.37 (0.29, 0.44) | <0.0001 |
| 2 | 0.25 (0.18, 0.33) | <0.0001 |
| 3 | 0.18 (0.10, 0.26) | <0.0001 |
| 4 | 0.11 (0.03, 0.19) | 0.0055 |
| 5 (highest) | Reference |  |
| Hospital variables |  | |
| Groups according to Hospital volume of pregnancy |  |  |
| 1 (lowest volume) | 0.67 (0.29, 1.05) | <0.0001 |
| 2 | Reference |  |
| 3 | 0.30 (- 0.01, 0.61) | 0.0597 |
| 4 | 0.36 (0.04, 0.69) | 0.0253 |
| 5 (highest volume) | 0.20 (- 0.11, 0.52) | 0.2031 |
| Province |  |  |
| Newfoundland and Labrador | 0.29 (- 0.16, 0.74) | 0.2110 |
| Prince Edward Island | - 0.95 (- 2.00, 0.10) | 0.0764 |
| Nova Scotia | - 0.29 (- 0.77, 0.19) | 0.2434 |
| New Brunswick | - 0.33 (- 0.79, 0.13) | 0.1569 |
| Ontario | Reference |  |
| Manitoba | - 1.08 (- 1.50, - 0.66) | <0.0001 |
| Saskatchewan | - 0.47 (- 0.84, - 0.09) | 0.0142 |
| Alberta | - 0.95 (- 1.22, - 0.67) | <0.0001 |
| British Columbia | - 0.68 (- 0.94, - 0.42) | <0.0001 |
| Territories | - 0.86 (- 2.07, 0.34) | 0.1582 |
| Hospital (Urban versus rural) | 0.25 (- 0.07, 0.52) | 0.1292 |
|  |  |  |
| Variance of random effects | 0.4642 | |
| Variance partition coefficient | 0.12365 | |
| Median odds ratio | 1.92 | |
