# Supplementary material for: Variability in intensive care unit admission among pregnant and postpartum women in Canada: a nationwide population-based observational study
Source: Crit Care. 2019 Nov 27;23:381. doi: 10.1186/s13054-019-2660-x (PMC6881971; doi:10.1186/s13054-019-2660-x)
Supplement: Supplementary file 11 — Additional file 11: Table S11. Sensitivity analysis: Estimated regression coefficients and variance components for the multi-level mixed logistic regression models for the outcome of ICU admission within multiple imputation datasets [Outcome=ICU admission, main predictors=quintile of hospitals according to the number of pregnancy admission at each hospital]. [file 13054_2019_2660_MOESM11_ESM.docx]

Table S11. Sensitivity analysis: Estimated regression coefficients and variance components for the multi-level mixed logistic regression models for the outcome of Intensive care unit (ICU) admission within multiple imputation datasets [Outcome=ICU admission, main predictors= quintile of hospitals according to the number of pregnancy admission at each hospital]

| Variable | Model 3 for ICU admission with Hospital group according to Hospital pregnancy volume | |
| --- | --- | --- |
|  | Regression coefficient (95% CI) | P-value |
| Intercept | - 6.51 (- 6.87, - 6.14) | <0.0001 |
| Patient variables |  | |
| Maternal Comorbidity Index | 0.63 (0.62, 0.64) | <0.0001 |
| Age, mean years |  |  |
| < 15 | 0.73 (0.02, 1.44) |  |
| 15-19 | 0.10 (0.00, 0.21) |  |
| 20-24 | Reference |  |
| 25-29 | 0.02 (- 0.04, 0.09) |  |
| 30-34 | 0.16 (0.10, 0.23) |  |
| 35-39 | 0.41 (0.34, 0.49) |  |
| 40-44 | 0.80 (0.70, 0.90) |  |
| > 44 | 1.06 (0.82, 1.31) |  |
| Parity | - 0.24 (- 0.27, - 0.21) | <0.0001 |
| Residence (urban versus rural) | 0.05 (- 0.01, 0.12) | 0.0960 |
| Transfer | 2.56 (2.49, 2.62) | <0.0001 |
| Income quintile |  |  |
| 1 (lowest) | 0.34 (0.27, 0.41) | <0.0001 |
| 2 | 0.25 (0.19, 0.35) | <0.0001 |
| 3 | 0.17 (0.10, 0.27) | <0.0001 |
| 4 | 0.10 (0.04, 0.19) | 0.0063 |
| 5 (highest) | Reference |  |
| Hospital variables |  | |
| Groups according to Hospital volume of pregnancy |  |  |
| 1 (lowest volume) | 0.73 (0.36, 1.1) | 0.0001 |
| 2 | Reference |  |
| 3 | 0.35 (0.04, 0.66) | 0.0262 |
| 4 | 0.48 (0.17, 0.79) | 0.0026 |
| 5 (highest volume) | 0.32 (0.01, 0.63) | 0.0414 |
| Province |  |  |
| Newfoundland and Labrador | 0.20 (- 0.25, 0.66) | 0.3810 |
| Prince Edward Island | - 0.73 (- 1.77, 0.30) | 0.1668 |
| Nova Scotia | - 0.35 (- 0.83, 0.12) | 0.1457 |
| New Brunswick | - 0.45 (- 0.92, 0.01) | 0.0573 |
| Ontario | Reference |  |
| Manitoba | - 1.15 (- 1.58, - 0.73) | <0.0001 |
| Saskatchewan | - 0.51 (- 0.89, - 0.14) | 0.0067 |
| Alberta | - 1.03 (- 1.30, - 0.76) | <0.0001 |
| British Columbia | - 0.73 (- 0.98, - 0.48) | <0.0001 |
| Territories | - 0.84 (- 1.72, 0.02) | 0.0562 |
| Hospital (Urban versus rural) | 0.23 (- 0.07, 0.52) | 0.1348 |
|  |  |  |
| Variance of random effects | 0.4795 | |
| Variance partition coefficient | 0.1272 | |
| Median odds ratio | 1.94 | |
