# Supplementary material for: Variability in intensive care unit admission among pregnant and postpartum women in Canada: a nationwide population-based observational study
Source: Crit Care. 2019 Nov 27;23:381. doi: 10.1186/s13054-019-2660-x (PMC6881971; doi:10.1186/s13054-019-2660-x)
Supplement: Supplementary file 12 — Additional file 12: Table S12. Search strategy in MEDLINE, EMBASE, Cochrane. [file 13054_2019_2660_MOESM12_ESM.docx]

Table S12. Search strategy in MEDLINE, EMBASE, Cochrane

**Medline**

Ovid MEDLINE(R) 1946 to September 24, 2019

| **#** | **Searches** | **Results** |
| --- | --- | --- |
| 1 | exp Intensive Care Units/ | 79168 |
| 2 | exp Critical Care/ | 55239 |
| 3 | Critical illness/ | 26635 |
| 4 | Critical Care Nursing/ | 1790 |
| 5 | exp Respiration, Artificial/ | 73910 |
| 6 | exp Ventilators, Mechanical/ | 8910 |
| 7 | Anesthesia Recovery Period/ | 5074 |
| 8 | "Delayed Emergence from Anesthesia"/ | 110 |
| 9 | "burn department*".mp. | 46 |
| 10 | "burn unit?".mp. | 3247 |
| 11 | "burn ward?".mp. | 107 |
| 12 | "coronary care department*".mp. | 8 |
| 13 | "coronary care unit?".mp. | 6808 |
| 14 | "coronary care ward?".mp. | 5 |
| 15 | "postacute care".mp. | 443 |
| 16 | "post-acute care".mp. | 643 |
| 17 | (an?esthe* adj2 recover*).mp. | 7203 |
| 18 | (critical?? adj2 ill*).mp,kw. | 49102 |
| 19 | (high adj1 acuity).mp. | 652 |
| 20 | (high* adj2 depend* adj2 department*).mp. | 6 |
| 21 | (high* adj2 depend* adj2 unit*).mp. | 561 |
| 22 | (high* adj2 depend* adj2 ward*).mp. | 36 |
| 23 | (intens* adj2 therap* adj2 department*).mp. | 45 |
| 24 | (intens* adj2 therap* adj2 unit*).mp. | 746 |
| 25 | (intens* adj2 therap* adj2 ward*).mp. | 37 |
| 26 | (neuro* adj2 intensive care?).mp. | 1634 |
| 27 | (neurointens* adj2 care?).mp. | 536 |
| 28 | (neuro-intens* adj2 care?).mp. | 160 |
| 29 | (postan?esth* adj2 care*).mp. | 1934 |
| 30 | (post-an?esth* adj2 care*).mp. | 879 |
| 31 | (special care adj2 unit?).mp. | 1104 |
| 32 | acute care setting?.mp. | 2613 |
| 33 | critical care.mp,jw. | 132774 |
| 34 | CTICU.mp. | 18 |
| 35 | CVICU.mp. | 48 |
| 36 | CVTICU.mp. | 0 |
| 37 | ICU.mp. | 42809 |
| 38 | ICUs.mp. | 7852 |
| 39 | IICU.mp. | 11 |
| 40 | intensive care?.mp,jw. | 156916 |
| 41 | intermediate care unit?.mp. | 434 |
| 42 | medsurg icu?.mp. | 0 |
| 43 | MICU.mp. | 600 |
| 44 | MSICU.mp. | 9 |
| 45 | neurocritical care?.mp,jw. | 2554 |
| 46 | neuro-critical care?.mp,jw. | 42 |
| 47 | NeuroICU.mp. | 16 |
| 48 | Neuro-ICU.mp. | 104 |
| 49 | progressive care unit?.mp. | 70 |
| 50 | teleICU.mp. | 4 |
| 51 | teleintensive*.mp. | 4 |
| 52 | ventilat*.mp. | 163331 |
| 53 | or/1-52 [ Critical Care / Intensive Care ] | 439191 |
| 54 | exp Pregnancy/ | 870092 |
| 55 | exp Pregnancy Complications/ | 413590 |
| 56 | exp Apgar Score/ | 7553 |
| 57 | exp Breast Feeding/ | 36148 |
| 58 | exp Fetal Development/ | 89652 |
| 59 | exp Fetal Monitoring/ | 8530 |
| 60 | exp Fetal Therapies/ | 3872 |
| 61 | exp Labor Pain/ | 1088 |
| 62 | exp Labor, Obstetric/ | 45402 |
| 63 | exp Maternal Health Services/ | 46804 |
| 64 | exp Midwifery/ | 18711 |
| 65 | exp Milk, Human/ | 18548 |
| 66 | exp Parity/ | 24408 |
| 67 | exp Pelvimetry/ | 1333 |
| 68 | exp Perinatal Care/ | 9497 |
| 69 | exp Placenta/ | 64644 |
| 70 | exp Postpartum Period/ | 62820 |
| 71 | exp Pregnancy Tests/ | 4022 |
| 72 | exp Prenatal Diagnosis/ | 72035 |
| 73 | exp Umbilical Cord/ | 26552 |
| 74 | exp Uterine Monitoring/ | 279 |
| 75 | (breast adj (fed or feed*)).mp,kw. | 42130 |
| 76 | (hemolys* adj4 elevat* liver enzyme* adj4 lowered platelet*).mp,kw. | 0 |
| 77 | ((labor or laboring) and (birth* or baby or babies or infan* or child* or newborn* or pregn* or deliver* or stage?)).mp,kw. | 88269 |
| 78 | abortion?.mp,kw. | 86048 |
| 79 | abruptio placent*.mp,kw. | 2835 |
| 80 | antenatal*.mp,kw. | 31229 |
| 81 | antepart*.mp,kw. | 5339 |
| 82 | breastfe*.mp,kw. | 21788 |
| 83 | breech present*.mp,kw. | 3916 |
| 84 | (caesar* adj2 (section* or deliver???)).mp,kw. | 17417 |
| 85 | cephalopelvic disproport*.mp,kw. | 585 |
| 86 | (cesar* adj2 (section* or deliver???)).mp,kw. | 55797 |
| 87 | childbirth*.mp,kw. | 18617 |
| 88 | chorea gravidar*.mp,kw. | 78 |
| 89 | chorioamnioniti*.mp,kw. | 4403 |
| 90 | dystocia?.mp,kw. | 5070 |
| 91 | eclamp*.mp,kw. | 36218 |
| 92 | episiotom*.mp,kw. | 3039 |
| 93 | gestational.mp,kw. | 147352 |
| 94 | hellp syndrome?.mp,kw. | 2385 |
| 95 | hyperemesis gravidar*.mp,kw. | 1842 |
| 96 | intrapart*.mp,kw. | 8823 |
| 97 | lactati*.mp,kw. | 60091 |
| 98 | matern*.mp,kw. | 292257 |
| 99 | morning sickness*.mp,kw. | 362 |
| 100 | nuchal cord?.mp,kw. | 264 |
| 101 | obstetric*.mp,kw. | 167229 |
| 102 | oligohydramni*.mp,kw. | 2686 |
| 103 | parturi*.mp,kw. | 26804 |
| 104 | perinatal*.mp,kw. | 67030 |
| 105 | placenta accreta*.mp,kw. | 2345 |
| 106 | placenta previa*.mp,kw. | 3638 |
| 107 | placenta*.mp,kw. | 106822 |
| 108 | postnatal*.mp,kw. | 101256 |
| 109 | postpart*.mp,kw. | 60969 |
| 110 | postpartum h?emorrhag*.mp,kw. | 8314 |
| 111 | preeclamp*.mp,kw. | 17732 |
| 112 | pre-eclamp*.mp,kw. | 32399 |
| 113 | pregnan*.mp,kw. | 927208 |
| 114 | premature birth*.mp,kw. | 15398 |
| 115 | prenatal*.mp,kw. | 160091 |
| 116 | prepartum.mp,kw. | 2015 |
| 117 | pre-partum.mp,kw. | 297 |
| 118 | puerper*.mp,kw. | 23147 |
| 119 | stillbirth*.mp,kw. | 11762 |
| 120 | still-birth*.mp,kw. | 644 |
| 121 | trimester?.mp,kw. | 66847 |
| 122 | trimestre?.mp,kw. | 798 |
| 123 | uterine inertia.mp,kw. | 994 |
| 124 | uterine inver*.mp,kw. | 444 |
| 125 | uterine ruptur*.mp,kw. | 4836 |
| 126 | vasa previa*.mp,kw. | 220 |
| 127 | or/54-126 [ Pregnancy or Partum ] | 1325196 |
| 128 | 53 and 127 [ ICU + Pregnancy ] | 29735 |
| 129 | Patient Admission/ | 23346 |
| 130 | Patient Selection/ | 62469 |
| 131 | (patient?? adj3 (admission* or admit*)).mp. | 100262 |
| 132 | (accept* adj3 patient??).mp. | 59980 |
| 133 | (patient?? adj2 present???).mp. | 167736 |
| 134 | (hospital?? adj3 (admission* or admit*)).mp. | 77409 |
| 135 | (maternal adj2 near-miss??).mp. | 246 |
| 136 | "appointments and schedules"/ | 8670 |
| 137 | waiting lists/ | 11394 |
| 138 | eligibility determination/ | 6017 |
| 139 | Health Services Accessibility/ | 70296 |
| 140 | or/129-139 [ Patient Admission ] | 517262 |
| 141 | 128 and 140 [ ICU + Pregnancy + Admission ] | 1859 |
| 142 | limit 141 to humans | 1853 |
| 143 | limit 142 to (female or pregnancy) | 1547 |
| 144 | remove duplicates from 143 | 1544 |
| 145 | limit 144 to ("all infant (birth to 23 months)" or "all child (0 to 18 years)" or "newborn infant (birth to 1 month)" or "infant (1 to 23 months)" or "preschool child (2 to 5 years)" or "child (6 to 12 years)" or "adolescent (13 to 18 years)") | 985 |
| 146 | 144 not 145 | 559 |
| 147 | limit 144 to ("all adult (19 plus years)" or "young adult (19 to 24 years)" or "adult (19 to 44 years)" or "young adult and adult (19-24 and 19-44)" or "middle age (45 to 64 years)" or "middle aged (45 plus years)" or "all aged (65 and over)" or "aged (80 and over)") | 919 |
| 148 | 146 or 147 | 1067 |
| 149 | limit 148 to english language | 936 |
| 150 | exp Clinical Audit/ | 21825 |
| 151 | sn.fs. [ Statistical & Numerical Data ] | 876617 |
| 152 | statistics as topic/ or statistics, nonparametric/ or exp survival analysis/ | 436456 |
| 153 | (audit or audits or audited or auditted or auditing or auditings).mp. | 46432 |
| 154 | statistic*.mp. | 1886388 |
| 155 | (data or datum).mp. | 4233062 |
| 156 | National Health Programs/ | 31374 |
| 157 | Government Programs/ | 5063 |
| 158 | national health program*.mp. | 31622 |
| 159 | national health service?.mp. | 11842 |
| 160 | (government???? adj2 program*).mp. | 6526 |
| 161 | provincial program???.mp. | 54 |
| 162 | provincial health*.mp. | 695 |
| 163 | nation-wide.mp. | 3030 |
| 164 | state-wide.mp. | 1122 |
| 165 | province-wide.mp. | 504 |
| 166 | big data/ | 505 |
| 167 | databases as topic/ | 9072 |
| 168 | databases, factual/ | 74445 |
| 169 | Dataset/ | 717 |
| 170 | datasets as topic/ | 3212 |
| 171 | data mining/ | 7692 |
| 172 | data warehousing/ | 88 |
| 173 | geographic information systems/ | 7489 |
| 174 | health information systems/ | 1175 |
| 175 | (nation?? adj3 database?).mp. | 11722 |
| 176 | (government?? adj3 database?).mp. | 224 |
| 177 | (state?? adj3 database?).mp. | 1505 |
| 178 | (provincial??? adj3 database?).mp. | 378 |
| 179 | "Information Storage and Retrieval"/ | 19567 |
| 180 | multicenter study/ | 257143 |
| 181 | Multicenter Studies as Topic/ | 17800 |
| 182 | ((multi-centre? or multi-center?) adj2 (study or studies)).mp. | 5030 |
| 183 | ((multicentre? or multicenter?) adj2 (study or studies)).mp. | 282835 |
| 184 | (international adj3 database?).mp. | 2487 |
| 185 | Registries/ | 82832 |
| 186 | (register? or registries).mp. | 140879 |
| 187 | (maternal adj3 audit?).mp. | 101 |
| 188 | (population* adj3 (study or studies)).mp. | 147859 |
| 189 | population surveillance/ or public health surveillance/ or sentinel surveillance/ | 65927 |
| 190 | (maternal adj3 surveillanc*).mp. | 292 |
| 191 | (nation?? adj3 surveillance).mp. | 6877 |
| 192 | (government* adj3 surveillance).mp. | 102 |
| 193 | (provinc* adj3 surveillance).mp. | 179 |
| 194 | ((state or states) adj3 surveillance).mp. | 1228 |
| 195 | *Hospitalization/ | 37652 |
| 196 | "national institutes of health (u.s.)"/ or "national institute of child health and human development (u.s.)"/ or "national institute of general medical sciences (u.s.)"/ or government/ or federal government/ or united states government agencies/ or exp "united states dept. of health and human services"/ or state government/ or government agencies/ or health planning organizations/ or "state health planning and development agencies"/ or health systems agencies/ or international agencies/ or world health organization/ or pan american health organization/ or state medicine/ | 219432 |
| 197 | (multicountry adj2 survey?).mp. | 46 |
| 198 | (multi-country adj2 survey?).mp. | 41 |
| 199 | Risk Assessment/ | 248210 |
| 200 | Patient Transfer/ | 7935 |
| 201 | Triage/ | 10932 |
| 202 | ep.fs. [Epidemiology] | 1588908 |
| 203 | or/150-202 | 6490623 |
| 204 | 149 and 203 | 682 |
| 205 | case reports.pt. | 1953218 |
| 206 | letter.pt. | 994665 |
| 207 | comment.pt. | 752622 |
| 208 | or/205-207 | 3073131 |
| 209 | 204 not 208 | 654 |
| 210 | remove duplicates from 209 | 654 |
| 211 | Neonatal Intensive Care?.ti. | 4279 |
| 212 | NICU.ti. | 1301 |
| 213 | NICUs.ti. | 97 |
| 214 | or/211-213 | 5626 |
| 215 | 210 not 214 | 622 |

**Medline In-Process / ePubs Ahead of Print**

Ovid MEDLINE(R) Epub Ahead of Print and In-Process & Other Non-Indexed Citations September 24, 2019

| **#** | **Searches** | **Results** |
| --- | --- | --- |
| 1 | exp Intensive Care Units/ | 0 |
| 2 | exp Critical Care/ | 0 |
| 3 | Critical illness/ | 0 |
| 4 | Critical Care Nursing/ | 0 |
| 5 | exp Respiration, Artificial/ | 0 |
| 6 | exp Ventilators, Mechanical/ | 0 |
| 7 | Anesthesia Recovery Period/ | 0 |
| 8 | "Delayed Emergence from Anesthesia"/ | 0 |
| 9 | "burn department*".mp. | 7 |
| 10 | "burn unit?".mp. | 186 |
| 11 | "burn ward?".mp. | 23 |
| 12 | "coronary care department*".mp. | 0 |
| 13 | "coronary care unit?".mp. | 238 |
| 14 | "coronary care ward?".mp. | 3 |
| 15 | "postacute care".mp. | 90 |
| 16 | "post-acute care".mp. | 191 |
| 17 | (an?esthe* adj2 recover*).mp. | 349 |
| 18 | (critical?? adj2 ill*).mp,kw. | 6909 |
| 19 | (high adj1 acuity).mp. | 139 |
| 20 | (high* adj2 depend* adj2 department*).mp. | 1 |
| 21 | (high* adj2 depend* adj2 unit*).mp. | 126 |
| 22 | (high* adj2 depend* adj2 ward*).mp. | 10 |
| 23 | (intens* adj2 therap* adj2 department*).mp. | 0 |
| 24 | (intens* adj2 therap* adj2 unit*).mp. | 65 |
| 25 | (intens* adj2 therap* adj2 ward*).mp. | 2 |
| 26 | (neuro* adj2 intensive care?).mp. | 243 |
| 27 | (neurointens* adj2 care?).mp. | 111 |
| 28 | (neuro-intens* adj2 care?).mp. | 42 |
| 29 | (postan?esth* adj2 care*).mp. | 335 |
| 30 | (post-an?esth* adj2 care*).mp. | 306 |
| 31 | (special care adj2 unit?).mp. | 84 |
| 32 | acute care setting?.mp. | 429 |
| 33 | critical care.mp,jw. | 11471 |
| 34 | CTICU.mp. | 3 |
| 35 | CVICU.mp. | 9 |
| 36 | CVTICU.mp. | 0 |
| 37 | ICU.mp. | 8882 |
| 38 | ICUs.mp. | 1571 |
| 39 | IICU.mp. | 3 |
| 40 | intensive care?.mp,jw. | 21038 |
| 41 | intermediate care unit?.mp. | 68 |
| 42 | medsurg icu?.mp. | 0 |
| 43 | MICU.mp. | 110 |
| 44 | MSICU.mp. | 4 |
| 45 | neurocritical care?.mp,jw. | 672 |
| 46 | neuro-critical care?.mp,jw. | 19 |
| 47 | NeuroICU.mp. | 7 |
| 48 | Neuro-ICU.mp. | 35 |
| 49 | progressive care unit?.mp. | 11 |
| 50 | teleICU.mp. | 2 |
| 51 | teleintensive*.mp. | 1 |
| 52 | ventilat*.mp. | 15438 |
| 53 | or/1-52 [ Critical Care / Intensive Care ] | 45730 |
| 54 | exp Pregnancy/ | 0 |
| 55 | exp Pregnancy Complications/ | 0 |
| 56 | exp Apgar Score/ | 0 |
| 57 | exp Breast Feeding/ | 0 |
| 58 | exp Fetal Development/ | 0 |
| 59 | exp Fetal Monitoring/ | 0 |
| 60 | exp Fetal Therapies/ | 0 |
| 61 | exp Labor Pain/ | 0 |
| 62 | exp Labor, Obstetric/ | 0 |
| 63 | exp Maternal Health Services/ | 0 |
| 64 | exp Midwifery/ | 0 |
| 65 | exp Milk, Human/ | 0 |
| 66 | exp Parity/ | 0 |
| 67 | exp Pelvimetry/ | 0 |
| 68 | exp Perinatal Care/ | 0 |
| 69 | exp Placenta/ | 0 |
| 70 | exp Postpartum Period/ | 0 |
| 71 | exp Pregnancy Tests/ | 0 |
| 72 | exp Prenatal Diagnosis/ | 0 |
| 73 | exp Umbilical Cord/ | 0 |
| 74 | exp Uterine Monitoring/ | 0 |
| 75 | (breast adj (fed or feed*)).mp,kw. | 1042 |
| 76 | (hemolys* adj4 elevat* liver enzyme* adj4 lowered platelet*).mp,kw. | 0 |
| 77 | ((labor or laboring) and (birth* or baby or babies or infan* or child* or newborn* or pregn* or deliver* or stage?)).mp,kw. | 3652 |
| 78 | abortion?.mp,kw. | 4197 |
| 79 | abruptio placent*.mp,kw. | 73 |
| 80 | antenatal*.mp,kw. | 4664 |
| 81 | antepart*.mp,kw. | 470 |
| 82 | breastfe*.mp,kw. | 5467 |
| 83 | breech present*.mp,kw. | 208 |
| 84 | (caesar* adj2 (section* or deliver???)).mp,kw. | 2918 |
| 85 | cephalopelvic disproport*.mp,kw. | 49 |
| 86 | (cesar* adj2 (section* or deliver???)).mp,kw. | 4026 |
| 87 | childbirth*.mp,kw. | 2275 |
| 88 | chorea gravidar*.mp,kw. | 6 |
| 89 | chorioamnioniti*.mp,kw. | 412 |
| 90 | dystocia?.mp,kw. | 351 |
| 91 | eclamp*.mp,kw. | 1915 |
| 92 | episiotom*.mp,kw. | 308 |
| 93 | gestational.mp,kw. | 11896 |
| 94 | hellp syndrome?.mp,kw. | 281 |
| 95 | hyperemesis gravidar*.mp,kw. | 161 |
| 96 | intrapart*.mp,kw. | 1242 |
| 97 | lactati*.mp,kw. | 4462 |
| 98 | matern*.mp,kw. | 29872 |
| 99 | morning sickness*.mp,kw. | 31 |
| 100 | nuchal cord?.mp,kw. | 22 |
| 101 | obstetric*.mp,kw. | 10737 |
| 102 | oligohydramni*.mp,kw. | 227 |
| 103 | parturi*.mp,kw. | 2017 |
| 104 | perinatal*.mp,kw. | 6871 |
| 105 | placenta accreta*.mp,kw. | 263 |
| 106 | placenta previa*.mp,kw. | 281 |
| 107 | placenta*.mp,kw. | 7357 |
| 108 | postnatal*.mp,kw. | 8466 |
| 109 | postpart*.mp,kw. | 6447 |
| 110 | postpartum h?emorrhag*.mp,kw. | 800 |
| 111 | preeclamp*.mp,kw. | 3091 |
| 112 | pre-eclamp*.mp,kw. | 1191 |
| 113 | pregnan*.mp,kw. | 46771 |
| 114 | premature birth*.mp,kw. | 473 |
| 115 | prenatal*.mp,kw. | 9838 |
| 116 | prepartum.mp,kw. | 144 |
| 117 | pre-partum.mp,kw. | 41 |
| 118 | puerper*.mp,kw. | 1356 |
| 119 | stillbirth*.mp,kw. | 1202 |
| 120 | still-birth*.mp,kw. | 94 |
| 121 | trimester?.mp,kw. | 5290 |
| 122 | trimestre?.mp,kw. | 58 |
| 123 | uterine inertia.mp,kw. | 11 |
| 124 | uterine inver*.mp,kw. | 60 |
| 125 | uterine ruptur*.mp,kw. | 335 |
| 126 | vasa previa*.mp,kw. | 25 |
| 127 | or/54-126 [ Pregnancy or Partum ] | 99846 |
| 128 | 53 and 127 [ ICU + Pregnancy ] | 3043 |
| 129 | Patient Admission/ | 0 |
| 130 | Patient Selection/ | 0 |
| 131 | (patient?? adj3 (admission* or admit*)).mp. | 13576 |
| 132 | (accept* adj3 patient??).mp. | 2664 |
| 133 | (patient?? adj2 present???).mp. | 29659 |
| 134 | (hospital?? adj3 (admission* or admit*)).mp. | 10717 |
| 135 | (maternal adj2 near-miss??).mp. | 65 |
| 136 | "appointments and schedules"/ | 0 |
| 137 | waiting lists/ | 0 |
| 138 | eligibility determination/ | 0 |
| 139 | Health Services Accessibility/ | 0 |
| 140 | or/129-139 [ Patient Admission ] | 52720 |
| 141 | 128 and 140 [ ICU + Pregnancy + Admission ] | 242 |
| 142 | exp Clinical Audit/ | 0 |
| 143 | sn.fs. [ Statistical & Numerical Data ] | 2 |
| 144 | statistics as topic/ or statistics, nonparametric/ or exp survival analysis/ | 0 |
| 145 | (audit or audits or audited or auditted or auditing or auditings).mp. | 5711 |
| 146 | statistic*.mp. | 164062 |
| 147 | (data or datum).mp. | 523247 |
| 148 | National Health Programs/ | 0 |
| 149 | Government Programs/ | 0 |
| 150 | national health program*.mp. | 90 |
| 151 | national health service?.mp. | 1741 |
| 152 | (government???? adj2 program*).mp. | 226 |
| 153 | provincial program???.mp. | 13 |
| 154 | provincial health*.mp. | 132 |
| 155 | nation-wide.mp. | 437 |
| 156 | state-wide.mp. | 206 |
| 157 | province-wide.mp. | 96 |
| 158 | big data/ | 0 |
| 159 | databases as topic/ | 0 |
| 160 | databases, factual/ | 0 |
| 161 | Dataset/ | 16 |
| 162 | datasets as topic/ | 0 |
| 163 | data mining/ | 0 |
| 164 | data warehousing/ | 0 |
| 165 | geographic information systems/ | 0 |
| 166 | health information systems/ | 0 |
| 167 | (nation?? adj3 database?).mp. | 3232 |
| 168 | (government?? adj3 database?).mp. | 53 |
| 169 | (state?? adj3 database?).mp. | 340 |
| 170 | (provincial??? adj3 database?).mp. | 70 |
| 171 | "Information Storage and Retrieval"/ | 0 |
| 172 | multicenter study/ | 4 |
| 173 | Multicenter Studies as Topic/ | 0 |
| 174 | ((multi-centre? or multi-center?) adj2 (study or studies)).mp. | 926 |
| 175 | ((multicentre? or multicenter?) adj2 (study or studies)).mp. | 6836 |
| 176 | (international adj3 database?).mp. | 637 |
| 177 | Registries/ | 0 |
| 178 | (register? or registries).mp. | 11700 |
| 179 | (maternal adj3 audit?).mp. | 17 |
| 180 | (population* adj3 (study or studies)).mp. | 24273 |
| 181 | population surveillance/ or public health surveillance/ or sentinel surveillance/ | 0 |
| 182 | (maternal adj3 surveillanc*).mp. | 40 |
| 183 | (nation?? adj3 surveillance).mp. | 1008 |
| 184 | (government* adj3 surveillance).mp. | 22 |
| 185 | (provinc* adj3 surveillance).mp. | 28 |
| 186 | ((state or states) adj3 surveillance).mp. | 103 |
| 187 | *Hospitalization/ | 0 |
| 188 | "national institutes of health (u.s.)"/ or "national institute of child health and human development (u.s.)"/ or "national institute of general medical sciences (u.s.)"/ or government/ or federal government/ or united states government agencies/ or exp "united states dept. of health and human services"/ or state government/ or government agencies/ or health planning organizations/ or "state health planning and development agencies"/ or health systems agencies/ or international agencies/ or world health organization/ or pan american health organization/ or state medicine/ | 0 |
| 189 | (multicountry adj2 survey?).mp. | 6 |
| 190 | (multi-country adj2 survey?).mp. | 15 |
| 191 | Risk Assessment/ | 0 |
| 192 | Patient Transfer/ | 0 |
| 193 | Triage/ | 0 |
| 194 | ep.fs. [Epidemiology] | 0 |
| 195 | or/142-194 | 654666 |
| 196 | 141 and 195 | 95 |
| 197 | limit 196 to english language | 94 |
| 198 | remove duplicates from 197 | 94 |

**Embase**

Embase Classic+Embase 1947 to 2019 September 25

| **#** | **Searches** | **Results** |
| --- | --- | --- |
| 1 | *catastrophic illness/ | 1687 |
| 2 | acute disease/ | 102802 |
| 3 | anesthetic recovery/ | 7709 |
| 4 | burn unit/ | 1483 |
| 5 | coronary care unit/ | 11350 |
| 6 | exp Intensive Care Units/ | 178880 |
| 7 | exp Critical Care/ | 690244 |
| 8 | intensive care nursing/ | 2044 |
| 9 | intensive care nursing/ | 2044 |
| 10 | intensive care unit/ | 146132 |
| 11 | intensivist/ | 3130 |
| 12 | medical intensive care unit/ | 1773 |
| 13 | neurological intensive care unit/ | 1138 |
| 14 | psychiatric intensive care unit/ | 49 |
| 15 | stroke unit/ | 5023 |
| 16 | surgical intensive care unit/ | 1307 |
| 17 | exp Intensive Care Units/ | 178880 |
| 18 | exp Critical Care/ | 690244 |
| 19 | Critical illness/ | 28066 |
| 20 | Critical Care Nursing/ | 1727 |
| 21 | exp Respiration, Artificial/ | 201142 |
| 22 | exp Ventilators, Mechanical/ | 3144 |
| 23 | Anesthesia Recovery Period/ | 7491 |
| 24 | "Delayed Emergence from Anesthesia"/ | 190 |
| 25 | "burn department*".mp. | 76 |
| 26 | "burn unit?".mp. | 2786 |
| 27 | "burn ward?".mp. | 158 |
| 28 | "coronary care department*".mp. | 17 |
| 29 | "coronary care unit?".mp. | 14026 |
| 30 | "coronary care ward?".mp. | 13 |
| 31 | "postacute care".mp. | 623 |
| 32 | "post-acute care".mp. | 1395 |
| 33 | "recovery department*".mp. | 26 |
| 34 | "recovery unit*".mp. | 687 |
| 35 | "recovery ward*".mp. | 169 |
| 36 | (an?esthe* adj2 recover*).mp. | 10744 |
| 37 | (critical?? adj2 ill*).mp,kw. | 90000 |
| 38 | (high adj1 acuity).mp. | 1343 |
| 39 | (high* adj2 depend* adj2 department*).mp. | 7 |
| 40 | (high* adj2 depend* adj2 unit*).mp. | 1447 |
| 41 | (high* adj2 depend* adj2 ward*).mp. | 93 |
| 42 | (intens* adj2 therap* adj2 department*).mp. | 68 |
| 43 | (intens* adj2 therap* adj2 unit*).mp. | 1152 |
| 44 | (intens* adj2 therap* adj2 ward*).mp. | 66 |
| 45 | (neuro* adj2 intensive care?).mp. | 3912 |
| 46 | (neurointens* adj2 care?).mp. | 1084 |
| 47 | (neuro-intens* adj2 care?).mp. | 395 |
| 48 | (postan?esth* adj2 care*).mp. | 3272 |
| 49 | (post-an?esth* adj2 care*).mp. | 1933 |
| 50 | (special care adj2 unit?).mp. | 1582 |
| 51 | acute care setting?.mp. | 3976 |
| 52 | critical care.mp,jw. | 200773 |
| 53 | CTICU.mp. | 83 |
| 54 | CVICU.mp. | 208 |
| 55 | CVTICU.mp. | 0 |
| 56 | ICU.mp. | 107006 |
| 57 | ICUs.mp. | 15433 |
| 58 | IICU.mp. | 25 |
| 59 | intensive care?.mp,jw. | 357544 |
| 60 | intermediate care unit?.mp. | 864 |
| 61 | medsurg icu?.mp. | 2 |
| 62 | MICU.mp. | 2348 |
| 63 | MSICU.mp. | 37 |
| 64 | neurocritical care?.mp,jw. | 6373 |
| 65 | neuro-critical care?.mp,jw. | 172 |
| 66 | NeuroICU.mp. | 120 |
| 67 | Neuro-ICU.mp. | 433 |
| 68 | progressive care unit?.mp. | 138 |
| 69 | stepdown unit?.mp. | 124 |
| 70 | step-down unit?.mp. | 559 |
| 71 | teleICU.mp. | 19 |
| 72 | teleintensive*.mp. | 5 |
| 73 | ventilat*.mp. | 328545 |
| 74 | or/1-73 [ Critical Care / Intensive Care ] | 1251974 |
| 75 | exp Pregnancy/ [ Medline & Embase ] | 783541 |
| 76 | exp Pregnancy Complications/ | 140946 |
| 77 | exp Apgar Score/ [ Medline & Embase ] | 23563 |
| 78 | exp Breast Feeding/ [ Medline & Embase ] | 52283 |
| 79 | exp Fetal Development/ | 28320 |
| 80 | exp Fetal Monitoring/ | 14596 |
| 81 | exp Fetal Therapies/ | 587 |
| 82 | exp Fetus/ | 210351 |
| 83 | exp Heart Rate, Fetal/ | 11017 |
| 84 | exp Infant, Newborn/ | 601583 |
| 85 | exp Labor Pain/ [ Medline & Embase ] | 3102 |
| 86 | exp Labor, Obstetric/ | 40429 |
| 87 | exp Maternal Health Services/ | 1306 |
| 88 | exp Midwifery/ | 32043 |
| 89 | exp Milk, Human/ | 29055 |
| 90 | exp Pelvimetry/ [ Medline & Embase ] | 1213 |
| 91 | exp Perinatal Care/ [ Medline & Embase ] | 57672 |
| 92 | exp Placenta/ [ Medline & Embase ] | 86648 |
| 93 | exp Postpartum Period/ | 68753 |
| 94 | exp Pregnancy Tests/ | 6475 |
| 95 | exp Prenatal Diagnosis/ | 108120 |
| 96 | exp Umbilical Cord/ [ Medline & Embase ] | 71232 |
| 97 | exp Uterine Monitoring/ | 63 |
| 98 | (breast adj (fed or feed*)).mp,kw. | 60190 |
| 99 | (extraembryon* adj2 membran*).mp,kw. | 394 |
| 100 | (hemolys* adj4 elevat* liver enzyme* adj4 lowered platelet*).mp,kw. | 1 |
| 101 | ((labor or laboring) and (birth* or baby or babies or infan* or child* or newborn* or pregn* or deliver* or stage?)).mp,kw. | 124199 |
| 102 | (new adj born*).mp,kw. | 8153 |
| 103 | abortion?.mp,kw. | 131085 |
| 104 | abruptio placent*.mp,kw. | 2095 |
| 105 | antenatal*.mp,kw. | 52627 |
| 106 | antepart*.mp,kw. | 9168 |
| 107 | birth???.mp,kw. | 490558 |
| 108 | breastfe*.mp,kw. | 34174 |
| 109 | breech present*.mp,kw. | 6431 |
| 110 | (caesar* adj2 (section* or deliver???)).mp,kw. | 36611 |
| 111 | cephalopelvic disproport*.mp,kw. | 1188 |
| 112 | (cesar* adj2 (section* or deliver???)).mp,kw. | 108822 |
| 113 | childbirth*.mp,kw. | 35430 |
| 114 | chorea gravidar*.mp,kw. | 99 |
| 115 | chorioamnioniti*.mp,kw. | 8771 |
| 116 | dystocia?.mp,kw. | 9479 |
| 117 | eclamp*.mp,kw. | 28026 |
| 118 | embryo los???.mp,kw. | 430 |
| 119 | embryo??.mp,kw. | 441796 |
| 120 | episiotom*.mp,kw. | 5416 |
| 121 | fetal.mp,kw. | 327256 |
| 122 | fetus*.mp,kw. | 394402 |
| 123 | foetal.mp,kw. | 33972 |
| 124 | foetus*.mp,kw. | 22458 |
| 125 | gestational.mp,kw. | 219944 |
| 126 | hellp syndrome?.mp,kw. | 5528 |
| 127 | hyperemesis gravidar*.mp,kw. | 3249 |
| 128 | intrapart*.mp,kw. | 14733 |
| 129 | lactati*.mp,kw. | 74972 |
| 130 | matern*.mp,kw. | 421781 |
| 131 | morning sickness*.mp,kw. | 655 |
| 132 | neonat*.mp,kw. | 366694 |
| 133 | newborn*.mp,kw. | 729917 |
| 134 | nuchal cord?.mp,kw. | 526 |
| 135 | obstetric*.mp,kw. | 185806 |
| 136 | oligohydramni*.mp,kw. | 6159 |
| 137 | parturi*.mp,kw. | 28977 |
| 138 | perinatal*.mp,kw. | 141775 |
| 139 | placenta accreta*.mp,kw. | 4341 |
| 140 | placenta previa*.mp,kw. | 7579 |
| 141 | placenta*.mp,kw. | 173847 |
| 142 | postnatal*.mp,kw. | 150928 |
| 143 | postpart*.mp,kw. | 75854 |
| 144 | postpartum h#emorrhag*.mp,kw. | 3338 |
| 145 | preeclamp*.mp,kw. | 61659 |
| 146 | pre-eclamp*.mp,kw. | 17517 |
| 147 | pregnan*.mp,kw. | 1060308 |
| 148 | premature birth*.mp,kw. | 7198 |
| 149 | prenatal*.mp,kw. | 254900 |
| 150 | prepartum.mp,kw. | 2392 |
| 151 | pre-partum.mp,kw. | 426 |
| 152 | puerper*.mp,kw. | 62232 |
| 153 | stillbirth*.mp,kw. | 23727 |
| 154 | still-birth*.mp,kw. | 1514 |
| 155 | trimester?.mp,kw. | 107205 |
| 156 | trimestre?.mp,kw. | 1155 |
| 157 | uterine inertia.mp,kw. | 552 |
| 158 | uterine inver*.mp,kw. | 531 |
| 159 | uterine ruptur*.mp,kw. | 4636 |
| 160 | vasa previa*.mp,kw. | 480 |
| 161 | exp Pregnancy/ [ Embase specific terms start here ] | 783541 |
| 162 | exp pregnancy complication/ | 140946 |
| 163 | exp pregnancy diabetes mellitus/ | 35306 |
| 164 | pregnancy disorder/ | 7663 |
| 165 | exp pregnancy outcome/ | 55959 |
| 166 | exp pregnancy test/ | 6475 |
| 167 | breast milk/ | 29055 |
| 168 | exp fetoplacental unit/ | 3360 |
| 169 | exp fetus development/ | 28320 |
| 170 | exp fetus disease/ | 119749 |
| 171 | exp fetus growth/ | 14901 |
| 172 | exp fetus heart rate/ | 11017 |
| 173 | exp labor/ | 40429 |
| 174 | exp labor complication/ | 205937 |
| 175 | exp labor management/ | 1335 |
| 176 | exp labor onset/ | 2818 |
| 177 | exp labor stage/ | 7339 |
| 178 | exp midwife/ | 32043 |
| 179 | exp obstetric operation/ | 168665 |
| 180 | exp obstetric procedure/ | 469092 |
| 181 | exp obstetrical nursing/ | 2777 |
| 182 | exp obstetrician/ | 6555 |
| 183 | exp prenatal diagnosis/ | 108120 |
| 184 | exp uterine activity monitor/ | 5 |
| 185 | exp uterine activity monitoring/ | 63 |
| 186 | newborn/ | 601583 |
| 187 | obstetric patient/ | 2065 |
| 188 | puerperium/ | 42996 |
| 189 | exp "parameters concerning the fetus, newborn and pregnancy"/ | 378353 |
| 190 | or/75-189 [ Pregnancy or Partum ] | 2894219 |
| 191 | 74 and 190 [ ICU + Pregnancy ] | 163999 |
| 192 | hospital admission/ | 186345 |
| 193 | Patient Admission/ | 174885 |
| 194 | patient selection/ | 90761 |
| 195 | (patient?? adj3 (admission* or admit*)).mp. | 182647 |
| 196 | (accept* adj3 patient??).mp. | 34119 |
| 197 | (patient?? adj2 present???).mp. | 341745 |
| 198 | (hospital?? adj3 (admission* or admit*)).mp. | 278647 |
| 199 | (maternal adj2 near-miss??).mp. | 449 |
| 200 | (wait??? adj1 list?).mp. | 20452 |
| 201 | elegibilit*.mp. | 78 |
| 202 | eligibilit*.mp. | 49910 |
| 203 | (Service? adj2 Accessibil*).mp. | 1711 |
| 204 | or/192-203 [ Patient Admission ] | 897435 |
| 205 | 191 and 204 [ ICU + Pregnancy + Patient Admission ] | 13353 |
| 206 | clinical audit/ | 3874 |
| 207 | statistics/ or biostatistics/ | 333631 |
| 208 | survival/ or survival analysis/ or survival factor/ | 340802 |
| 209 | exp statistical analysis/ | 2261311 |
| 210 | public health/ | 179506 |
| 211 | government/ | 145736 |
| 212 | data analysis/ or data base/ or data warehouse/ or exp factual database/ | 426966 |
| 213 | data mining/ | 11954 |
| 214 | data warehouse/ | 874 |
| 215 | geographic information system/ | 9846 |
| 216 | medical information system/ | 20331 |
| 217 | information retrieval/ | 35993 |
| 218 | multicenter study/ | 231057 |
| 219 | "multicenter study (topic)"/ | 29672 |
| 220 | register/ | 114205 |
| 221 | *hospitalization/ | 34657 |
| 222 | health care organization/ or exp health care industry/ or exp health care system/ or exp hospital organization/ or medicaid/ or medical society/ or medicare/ or national health insurance/ or national health organization/ or national health service/ or exp non profit organization/ or world health organization/ | 1100279 |
| 223 | risk assessment/ or risk benefit analysis/ or "risk evaluation and mitigation strategy"/ | 578593 |
| 224 | hospital emergency service/ | 3886 |
| 225 | ep.fs. [Epidemiology] | 1046374 |
| 226 | (audit or audits or audited or auditted or auditing or auditings).mp. | 93670 |
| 227 | statistic*.mp. | 2224440 |
| 228 | (data or datum).mp. | 5203261 |
| 229 | national health program*.mp. | 724 |
| 230 | national health service?.mp. | 75894 |
| 231 | (government???? adj2 program*).mp. | 2613 |
| 232 | provincial program???.mp. | 118 |
| 233 | provincial health*.mp. | 1225 |
| 234 | nation-wide.mp. | 5942 |
| 235 | state-wide.mp. | 2381 |
| 236 | province-wide.mp. | 979 |
| 237 | (nation?? adj3 database?).mp. | 25685 |
| 238 | (government?? adj3 database?).mp. | 477 |
| 239 | (state?? adj3 database?).mp. | 2937 |
| 240 | (provincial??? adj3 database?).mp. | 825 |
| 241 | ((multi-centre? or multi-center?) adj2 (study or studies)).mp. | 13479 |
| 242 | ((multicentre? or multicenter?) adj2 (study or studies)).mp. | 292462 |
| 243 | (international adj3 database?).mp. | 4554 |
| 244 | (register? or registries).mp. | 206008 |
| 245 | (maternal adj3 audit?).mp. | 213 |
| 246 | (population* adj3 (study or studies)).mp. | 260871 |
| 247 | (maternal adj3 surveillanc*).mp. | 440 |
| 248 | (nation?? adj3 surveillance).mp. | 10106 |
| 249 | (government* adj3 surveillance).mp. | 150 |
| 250 | (provinc* adj3 surveillance).mp. | 282 |
| 251 | ((state or states) adj3 surveillance).mp. | 1610 |
| 252 | (multicountry adj2 survey?).mp. | 61 |
| 253 | (multi-country adj2 survey?).mp. | 95 |
| 254 | or/206-253 [ Data ] | 9889960 |
| 255 | 205 and 254 [ ICU + Pregnancy + Patient Admission + Data ] | 7444 |
| 256 | limit 255 to english language | 7074 |
| 257 | (exp animals/ or exp animal experimentation/ or nonhuman/) not ((exp animals/ or exp animal experimentation/ or nonhuman/) and exp human/) | 7110648 |
| 258 | 256 not 257 | 7054 |
| 259 | limit 256 to human | 6780 |
| 260 | 258 or 259 | 7054 |
| 261 | Neonatal Intensive Care?.ti. | 6234 |
| 262 | NICU.ti. | 2364 |
| 263 | NICUs.ti. | 186 |
| 264 | or/261-263 | 8571 |
| 265 | 260 not 264 | 6631 |
| 266 | case report/ | 2508301 |
| 267 | book/ or editorial/ or letter/ or note/ | 2434108 |
| 268 | 266 or 267 | 4676221 |
| 269 | 265 not 268 | 6252 |
| 270 | limit 269 to (conference abstract status or (books or chapter or conference abstract or "conference review") or (book or book series or conference proceeding)) | 1710 |
| 271 | 269 not 270 | 4542 |
| 272 | remove duplicates from 271 | 4412 |

**CCTR**

Cochrane Central Register of Controlled Trials 2014 to Present

| **#** | **Searches** | **Results** |
| --- | --- | --- |
| 1 | *catastrophic illness/ | 0 |
| 2 | acute disease/ | 9034 |
| 3 | anesthetic recovery/ | 0 |
| 4 | burn unit/ | 45 |
| 5 | coronary care unit/ | 144 |
| 6 | exp Intensive Care Units/ | 3284 |
| 7 | exp Critical Care/ | 1933 |
| 8 | intensive care nursing/ | 35 |
| 9 | intensive care nursing/ | 35 |
| 10 | intensive care unit/ | 1 |
| 11 | intensivist/ | 0 |
| 12 | medical intensive care unit/ | 0 |
| 13 | neurological intensive care unit/ | 0 |
| 14 | psychiatric intensive care unit/ | 0 |
| 15 | stroke unit/ | 0 |
| 16 | surgical intensive care unit/ | 0 |
| 17 | exp Intensive Care Units/ | 3284 |
| 18 | exp Critical Care/ | 1933 |
| 19 | Critical illness/ | 1861 |
| 20 | Critical Care Nursing/ | 35 |
| 21 | exp Respiration, Artificial/ | 5691 |
| 22 | exp Ventilators, Mechanical/ | 255 |
| 23 | Anesthesia Recovery Period/ | 1987 |
| 24 | "Delayed Emergence from Anesthesia"/ | 47 |
| 25 | "burn department*".mp. | 3 |
| 26 | "burn unit?".mp. | 130 |
| 27 | "burn ward?".mp. | 16 |
| 28 | "coronary care department*".mp. | 1 |
| 29 | "coronary care unit?".mp. | 640 |
| 30 | "coronary care ward?".mp. | 4 |
| 31 | "postacute care".mp. | 29 |
| 32 | "post-acute care".mp. | 104 |
| 33 | "recovery department*".mp. | 4 |
| 34 | "recovery unit*".mp. | 205 |
| 35 | "recovery ward*".mp. | 83 |
| 36 | (an?esthe* adj2 recover*).mp. | 4601 |
| 37 | (critical?? adj2 ill*).mp,kw. | 7396 |
| 38 | (high adj1 acuity).mp. | 76 |
| 39 | (high* adj2 depend* adj2 department*).mp. | 0 |
| 40 | (high* adj2 depend* adj2 unit*).mp. | 99 |
| 41 | (high* adj2 depend* adj2 ward*).mp. | 16 |
| 42 | (intens* adj2 therap* adj2 department*).mp. | 1 |
| 43 | (intens* adj2 therap* adj2 unit*).mp. | 374 |
| 44 | (intens* adj2 therap* adj2 ward*).mp. | 2 |
| 45 | (neuro* adj2 intensive care?).mp. | 288 |
| 46 | (neurointens* adj2 care?).mp. | 47 |
| 47 | (neuro-intens* adj2 care?).mp. | 19 |
| 48 | (postan?esth* adj2 care*).mp. | 1334 |
| 49 | (post-an?esth* adj2 care*).mp. | 1076 |
| 50 | (special care adj2 unit?).mp. | 155 |
| 51 | acute care setting?.mp. | 208 |
| 52 | critical care.mp,jw. | 7630 |
| 53 | CTICU.mp. | 5 |
| 54 | CVICU.mp. | 20 |
| 55 | CVTICU.mp. | 0 |
| 56 | ICU.mp. | 10954 |
| 57 | ICUs.mp. | 1421 |
| 58 | IICU.mp. | 5 |
| 59 | intensive care?.mp,jw. | 22895 |
| 60 | intermediate care unit?.mp. | 79 |
| 61 | medsurg icu?.mp. | 0 |
| 62 | MICU.mp. | 110 |
| 63 | MSICU.mp. | 1 |
| 64 | neurocritical care?.mp,jw. | 317 |
| 65 | neuro-critical care?.mp,jw. | 9 |
| 66 | NeuroICU.mp. | 4 |
| 67 | Neuro-ICU.mp. | 21 |
| 68 | progressive care unit?.mp. | 6 |
| 69 | stepdown unit?.mp. | 11 |
| 70 | step-down unit?.mp. | 50 |
| 71 | teleICU.mp. | 0 |
| 72 | teleintensive*.mp. | 0 |
| 73 | ventilat*.mp. | 29101 |
| 74 | or/1-73 [ Critical Care / Intensive Care ] | 68633 |
| 75 | exp Pregnancy/ [ Medline & Embase ] | 20068 |
| 76 | exp Pregnancy Complications/ | 10045 |
| 77 | exp Apgar Score/ [ Medline & Embase ] | 664 |
| 78 | exp Breast Feeding/ [ Medline & Embase ] | 1701 |
| 79 | exp Fetal Development/ | 2570 |
| 80 | exp Fetal Monitoring/ | 339 |
| 81 | exp Fetal Therapies/ | 36 |
| 82 | exp Fetus/ | 1627 |
| 83 | exp Heart Rate, Fetal/ | 328 |
| 84 | exp Infant, Newborn/ | 14626 |
| 85 | exp Labor Pain/ [ Medline & Embase ] | 308 |
| 86 | exp Labor, Obstetric/ | 2149 |
| 87 | exp Maternal Health Services/ | 1943 |
| 88 | exp Midwifery/ | 306 |
| 89 | exp Milk, Human/ | 937 |
| 90 | exp Pelvimetry/ [ Medline & Embase ] | 9 |
| 91 | exp Perinatal Care/ [ Medline & Embase ] | 503 |
| 92 | exp Placenta/ [ Medline & Embase ] | 340 |
| 93 | exp Postpartum Period/ | 1464 |
| 94 | exp Pregnancy Tests/ | 25 |
| 95 | exp Prenatal Diagnosis/ | 758 |
| 96 | exp Umbilical Cord/ [ Medline & Embase ] | 501 |
| 97 | exp Uterine Monitoring/ | 15 |
| 98 | (breast adj (fed or feed*)).mp,kw. | 4795 |
| 99 | (extraembryon* adj2 membran*).mp,kw. | 58 |
| 100 | (hemolys* adj4 elevat* liver enzyme* adj4 lowered platelet*).mp,kw. | 0 |
| 101 | ((labor or laboring) and (birth* or baby or babies or infan* or child* or newborn* or pregn* or deliver* or stage?)).mp,kw. | 9544 |
| 102 | (new adj born*).mp,kw. | 223 |
| 103 | abortion?.mp,kw. | 5213 |
| 104 | abruptio placent*.mp,kw. | 76 |
| 105 | antenatal*.mp,kw. | 3885 |
| 106 | antepart*.mp,kw. | 485 |
| 107 | birth???.mp,kw. | 26601 |
| 108 | breastfe*.mp,kw. | 4466 |
| 109 | breech present*.mp,kw. | 256 |
| 110 | (caesar* adj2 (section* or deliver???)).mp,kw. | 4191 |
| 111 | cephalopelvic disproport*.mp,kw. | 52 |
| 112 | (cesar* adj2 (section* or deliver???)).mp,kw. | 9766 |
| 113 | childbirth*.mp,kw. | 3062 |
| 114 | chorea gravidar*.mp,kw. | 0 |
| 115 | chorioamnioniti*.mp,kw. | 676 |
| 116 | dystocia?.mp,kw. | 396 |
| 117 | eclamp*.mp,kw. | 1948 |
| 118 | embryo los???.mp,kw. | 13 |
| 119 | embryo??.mp,kw. | 6366 |
| 120 | episiotom*.mp,kw. | 895 |
| 121 | fetal.mp,kw. | 10378 |
| 122 | fetus*.mp,kw. | 6036 |
| 123 | foetal.mp,kw. | 580 |
| 124 | foetus*.mp,kw. | 225 |
| 125 | gestational.mp,kw. | 15903 |
| 126 | hellp syndrome?.mp,kw. | 190 |
| 127 | hyperemesis gravidar*.mp,kw. | 157 |
| 128 | intrapart*.mp,kw. | 1090 |
| 129 | lactati*.mp,kw. | 4003 |
| 130 | matern*.mp,kw. | 20349 |
| 131 | morning sickness*.mp,kw. | 61 |
| 132 | neonat*.mp,kw. | 20300 |
| 133 | newborn*.mp,kw. | 25702 |
| 134 | nuchal cord?.mp,kw. | 8 |
| 135 | obstetric*.mp,kw. | 13539 |
| 136 | oligohydramni*.mp,kw. | 252 |
| 137 | parturi*.mp,kw. | 2488 |
| 138 | perinatal*.mp,kw. | 5244 |
| 139 | placenta accreta*.mp,kw. | 88 |
| 140 | placenta previa*.mp,kw. | 316 |
| 141 | placenta*.mp,kw. | 3736 |
| 142 | postnatal*.mp,kw. | 4167 |
| 143 | postpart*.mp,kw. | 8015 |
| 144 | postpartum h#emorrhag*.mp,kw. | 432 |
| 145 | preeclamp*.mp,kw. | 2434 |
| 146 | pre-eclamp*.mp,kw. | 1623 |
| 147 | pregnan*.mp,kw. | 58683 |
| 148 | premature birth*.mp,kw. | 1314 |
| 149 | prenatal*.mp,kw. | 6102 |
| 150 | prepartum.mp,kw. | 62 |
| 151 | pre-partum.mp,kw. | 14 |
| 152 | puerper*.mp,kw. | 2362 |
| 153 | stillbirth*.mp,kw. | 854 |
| 154 | still-birth*.mp,kw. | 75 |
| 155 | trimester?.mp,kw. | 5778 |
| 156 | trimestre?.mp,kw. | 36 |
| 157 | uterine inertia.mp,kw. | 48 |
| 158 | uterine inver*.mp,kw. | 10 |
| 159 | uterine ruptur*.mp,kw. | 160 |
| 160 | vasa previa*.mp,kw. | 9 |
| 161 | exp Pregnancy/ [ Embase specific terms start here ] | 20068 |
| 162 | exp pregnancy complication/ | 10045 |
| 163 | exp pregnancy diabetes mellitus/ | 0 |
| 164 | pregnancy disorder/ | 0 |
| 165 | exp pregnancy outcome/ | 2983 |
| 166 | exp pregnancy test/ | 25 |
| 167 | breast milk/ | 937 |
| 168 | exp fetoplacental unit/ | 0 |
| 169 | exp fetus development/ | 0 |
| 170 | exp fetus disease/ | 0 |
| 171 | exp fetus growth/ | 0 |
| 172 | exp fetus heart rate/ | 0 |
| 173 | exp labor/ | 0 |
| 174 | exp labor complication/ | 3264 |
| 175 | exp labor management/ | 0 |
| 176 | exp labor onset/ | 550 |
| 177 | exp labor stage/ | 0 |
| 178 | exp midwife/ | 306 |
| 179 | exp obstetric operation/ | 0 |
| 180 | exp obstetric procedure/ | 0 |
| 181 | exp obstetrical nursing/ | 43 |
| 182 | exp obstetrician/ | 0 |
| 183 | exp prenatal diagnosis/ | 758 |
| 184 | exp uterine activity monitor/ | 0 |
| 185 | exp uterine activity monitoring/ | 0 |
| 186 | newborn/ | 14496 |
| 187 | obstetric patient/ | 0 |
| 188 | puerperium/ | 1024 |
| 189 | exp "parameters concerning the fetus, newborn and pregnancy"/ | 0 |
| 190 | or/75-189 [ Pregnancy or Partum ] | 108338 |
| 191 | 74 and 190 [ ICU + Pregnancy ] | 9301 |
| 192 | hospital admission/ | 0 |
| 193 | Patient Admission/ | 566 |
| 194 | patient selection/ | 3366 |
| 195 | (patient?? adj3 (admission* or admit*)).mp. | 13897 |
| 196 | (accept* adj3 patient??).mp. | 10049 |
| 197 | (patient?? adj2 present???).mp. | 18798 |
| 198 | (hospital?? adj3 (admission* or admit*)).mp. | 14721 |
| 199 | (maternal adj2 near-miss??).mp. | 18 |
| 200 | (wait??? adj1 list?).mp. | 6666 |
| 201 | elegibilit*.mp. | 15 |
| 202 | eligibilit*.mp. | 12724 |
| 203 | (Service? adj2 Accessibil*).mp. | 667 |
| 204 | or/192-203 [ Patient Admission ] | 72153 |
| 205 | 191 and 204 [ ICU + Pregnancy + Patient Admission ] | 788 |
| 206 | clinical audit/ | 21 |
| 207 | statistics/ or biostatistics/ | 9 |
| 208 | survival/ or survival analysis/ or survival factor/ | 8130 |
| 209 | exp statistical analysis/ | 0 |
| 210 | public health/ | 215 |
| 211 | government/ | 8 |
| 212 | data analysis/ or data base/ or data warehouse/ or exp factual database/ | 367 |
| 213 | data mining/ | 17 |
| 214 | data warehouse/ | 0 |
| 215 | geographic information system/ | 38 |
| 216 | medical information system/ | 0 |
| 217 | information retrieval/ | 2 |
| 218 | multicenter study/ | 11 |
| 219 | "multicenter study (topic)"/ | 1 |
| 220 | register/ | 0 |
| 221 | *hospitalization/ | 0 |
| 222 | health care organization/ or exp health care industry/ or exp health care system/ or exp hospital organization/ or medicaid/ or medical society/ or medicare/ or national health insurance/ or national health organization/ or national health service/ or exp non profit organization/ or world health organization/ | 44388 |
| 223 | risk assessment/ or risk benefit analysis/ or "risk evaluation and mitigation strategy"/ | 8541 |
| 224 | hospital emergency service/ | 2004 |
| 225 | ep.fs. [Epidemiology] | 38497 |
| 226 | (audit or audits or audited or auditted or auditing or auditings).mp. | 3631 |
| 227 | statistic*.mp. | 198929 |
| 228 | (data or datum).mp. | 259667 |
| 229 | national health program*.mp. | 205 |
| 230 | national health service?.mp. | 1474 |
| 231 | (government???? adj2 program*).mp. | 167 |
| 232 | provincial program???.mp. | 3 |
| 233 | provincial health*.mp. | 39 |
| 234 | nation-wide.mp. | 218 |
| 235 | state-wide.mp. | 100 |
| 236 | province-wide.mp. | 30 |
| 237 | (nation?? adj3 database?).mp. | 841 |
| 238 | (government?? adj3 database?).mp. | 15 |
| 239 | (state?? adj3 database?).mp. | 103 |
| 240 | (provincial??? adj3 database?).mp. | 32 |
| 241 | ((multi-centre? or multi-center?) adj2 (study or studies)).mp. | 6073 |
| 242 | ((multicentre? or multicenter?) adj2 (study or studies)).mp. | 87214 |
| 243 | (international adj3 database?).mp. | 155 |
| 244 | (register? or registries).mp. | 16319 |
| 245 | (maternal adj3 audit?).mp. | 10 |
| 246 | (population* adj3 (study or studies)).mp. | 24003 |
| 247 | (maternal adj3 surveillanc*).mp. | 15 |
| 248 | (nation?? adj3 surveillance).mp. | 181 |
| 249 | (government* adj3 surveillance).mp. | 3 |
| 250 | (provinc* adj3 surveillance).mp. | 9 |
| 251 | ((state or states) adj3 surveillance).mp. | 28 |
| 252 | (multicountry adj2 survey?).mp. | 0 |
| 253 | (multi-country adj2 survey?).mp. | 2 |
| 254 | or/206-253 [ Data ] | 528578 |
| 255 | 205 and 254 [ ICU + Pregnancy + Patient Admission + Data ] | 438 |
| 256 | Neonatal Intensive Care?.ti. | 347 |
| 257 | NICU.ti. | 251 |
| 258 | NICUs.ti. | 13 |
| 259 | or/256-258 | 591 |
| 260 | 255 not 259 | 416 |
| 261 | limit 260 to english language | 306 |
| 262 | (abstract or book or book article or book book or book note or "book review" or book series article or book series article in press or book series chapter or book series conference paper or book series letter or "book series review" or book series short survey or chapter or conference abstract or conference abstract placebo controlled partly blinded crossover study in 12 sle patients or conference proceeding or "conference review" or journal conference abstract or "journal conference review").pt. | 156528 |
| 263 | 261 not 262 | 248 |
| 264 | remove duplicates from 263 | 240 |

**CDSR**

Cochrane Database of Systematic Reviews 2005 to Present

| **#** | **Searches** | **Results** |
| --- | --- | --- |
| 1 | "burn department*".ti,ab. | 0 |
| 2 | "burn unit?".ti,ab. | 0 |
| 3 | "burn ward?".ti,ab. | 0 |
| 4 | "coronary care department*".ti,ab. | 0 |
| 5 | "coronary care unit?".ti,ab. | 1 |
| 6 | "coronary care ward?".ti,ab. | 0 |
| 7 | "postacute care".ti,ab. | 0 |
| 8 | "post-acute care".ti,ab. | 0 |
| 9 | "recovery department*".ti,ab. | 0 |
| 10 | "recovery unit*".ti,ab. | 0 |
| 11 | "recovery ward*".ti,ab. | 1 |
| 12 | (an?esthe* adj2 recover*).ti,ab. | 5 |
| 13 | (critical?? adj2 ill*).ti,ab. | 93 |
| 14 | (high adj1 acuity).ti,ab. | 0 |
| 15 | (high* adj2 depend* adj2 department*).ti,ab. | 0 |
| 16 | (high* adj2 depend* adj2 unit*).ti,ab. | 4 |
| 17 | (high* adj2 depend* adj2 ward*).ti,ab. | 0 |
| 18 | (intens* adj2 therap* adj2 department*).ti,ab. | 0 |
| 19 | (intens* adj2 therap* adj2 unit*).ti,ab. | 4 |
| 20 | (intens* adj2 therap* adj2 ward*).ti,ab. | 0 |
| 21 | (neuro* adj2 intensive care?).ti,ab. | 0 |
| 22 | (neurointens* adj2 care?).ti,ab. | 0 |
| 23 | (neuro-intens* adj2 care?).ti,ab. | 0 |
| 24 | (postan?esth* adj2 care*).ti,ab. | 3 |
| 25 | (post-an?esth* adj2 care*).ti,ab. | 1 |
| 26 | (special care adj2 unit?).ti,ab. | 2 |
| 27 | acute care setting?.ti,ab. | 4 |
| 28 | critical care.mp,jw. | 274 |
| 29 | CTICU.ti,ab. | 0 |
| 30 | CVICU.ti,ab. | 0 |
| 31 | CVTICU.ti,ab. | 0 |
| 32 | ICU.ti,ab. | 91 |
| 33 | ICUs.ti,ab. | 23 |
| 34 | IICU.ti,ab. | 0 |
| 35 | intensive care?.ti,ab. | 194 |
| 36 | intermediate care unit?.ti,ab. | 1 |
| 37 | medsurg icu?.ti,ab. | 0 |
| 38 | MICU.ti,ab. | 0 |
| 39 | MSICU.ti,ab. | 0 |
| 40 | neurocritical care?.ti,ab. | 0 |
| 41 | neuro-critical care?.ti,ab. | 0 |
| 42 | NeuroICU.ti,ab. | 0 |
| 43 | Neuro-ICU.ti,ab. | 0 |
| 44 | progressive care unit?.ti,ab. | 0 |
| 45 | stepdown unit?.ti,ab. | 1 |
| 46 | step-down unit?.ti,ab. | 1 |
| 47 | teleICU.ti,ab. | 0 |
| 48 | teleintensive*.ti,ab. | 0 |
| 49 | ventilat*.ti,ab. | 290 |
| 50 | or/1-49 [ ICU / Critical Care ] | 622 |
| 51 | (breast adj (fed or feed*)).ti,ab. | 15 |
| 52 | (extraembryon* adj2 membran*).ti,ab. | 0 |
| 53 | (hemolys* adj4 elevat* liver enzyme* adj4 lowered platelet*).ti,ab. | 0 |
| 54 | ((labor or laboring) and (birth* or baby or babies or infan* or child* or newborn* or pregn* or deliver* or stage?)).ti,ab. | 13 |
| 55 | (new adj born*).ti,ab. | 0 |
| 56 | abortion?.ti,ab. | 24 |
| 57 | abruptio placent*.ti,ab. | 0 |
| 58 | antenatal*.ti,ab. | 122 |
| 59 | antepart*.ti,ab. | 19 |
| 60 | birth???.ti,ab. | 673 |
| 61 | breastfe*.ti,ab. | 72 |
| 62 | breech present*.ti,ab. | 11 |
| 63 | (caesar* adj2 (section* or deliver???)).ti,ab. | 155 |
| 64 | cephalopelvic disproport*.ti,ab. | 0 |
| 65 | (cesar* adj2 (section* or deliver???)).ti,ab. | 11 |
| 66 | childbirth*.ti,ab. | 603 |
| 67 | chorea gravidar*.ti,ab. | 0 |
| 68 | chorioamnioniti*.ti,ab. | 11 |
| 69 | dystocia?.ti,ab. | 7 |
| 70 | eclamp*.ti,ab. | 63 |
| 71 | embryo los???.ti,ab. | 1 |
| 72 | embryo??.ti,ab. | 38 |
| 73 | episiotom*.ti,ab. | 24 |
| 74 | fetal.ti,ab. | 196 |
| 75 | fetus*.ti,ab. | 75 |
| 76 | foetal.ti,ab. | 14 |
| 77 | foetus*.ti,ab. | 4 |
| 78 | gestational.ti,ab. | 203 |
| 79 | hellp syndrome?.ti,ab. | 3 |
| 80 | hyperemesis gravidar*.ti,ab. | 2 |
| 81 | intrapart*.ti,ab. | 26 |
| 82 | lactati*.ti,ab. | 32 |
| 83 | matern*.ti,ab. | 418 |
| 84 | morning sickness*.ti,ab. | 0 |
| 85 | neonat*.ti,ab. | 671 |
| 86 | newborn*.ti,ab. | 232 |
| 87 | nuchal cord?.ti,ab. | 0 |
| 88 | obstetric*.ti,ab. | 77 |
| 89 | oligohydramni*.ti,ab. | 8 |
| 90 | parturi*.ti,ab. | 6 |
| 91 | perinatal*.ti,ab. | 244 |
| 92 | placenta accreta*.ti,ab. | 0 |
| 93 | placenta previa*.ti,ab. | 0 |
| 94 | placenta*.ti,ab. | 64 |
| 95 | postnatal*.ti,ab. | 76 |
| 96 | postpart*.ti,ab. | 137 |
| 97 | postpartum h#emorrhag*.ti,ab. | 35 |
| 98 | preeclamp*.ti,ab. | 0 |
| 99 | pre-eclamp*.ti,ab. | 61 |
| 100 | pregnan*.ti,ab. | 964 |
| 101 | premature birth*.ti,ab. | 6 |
| 102 | prenatal*.ti,ab. | 41 |
| 103 | prepartum.ti,ab. | 0 |
| 104 | pre-partum.ti,ab. | 0 |
| 105 | puerper*.ti,ab. | 9 |
| 106 | stillbirth*.ti,ab. | 40 |
| 107 | still-birth*.ti,ab. | 0 |
| 108 | trimester?.ti,ab. | 69 |
| 109 | trimestre?.ti,ab. | 0 |
| 110 | uterine inertia.ti,ab. | 0 |
| 111 | uterine inver*.ti,ab. | 1 |
| 112 | uterine ruptur*.ti,ab. | 6 |
| 113 | vasa previa*.ti,ab. | 0 |
| 114 | or/51-113 [ Pregnancy ] | 1712 |
| 115 | 50 and 114 [ ICU + Pregnancy ] | 210 |
| 116 | (patient?? adj3 (admission* or admit*)).ti,ab. | 31 |
| 117 | (accept* adj3 patient??).ti,ab. | 24 |
| 118 | (patient?? adj2 present???).ti,ab. | 55 |
| 119 | (hospital?? adj3 (admission* or admit*)).ti,ab. | 188 |
| 120 | (maternal adj2 near-miss??).ti,ab. | 0 |
| 121 | (wait??? adj1 list?).ti,ab. | 126 |
| 122 | elegibilit*.ti,ab. | 0 |
| 123 | eligibilit*.ti,ab. | 918 |
| 124 | (Service? adj2 Accessibil*).ti,ab. | 2 |
| 125 | or/116-124 [ Admission ] | 1286 |
| 126 | 115 and 125 [ ICU + Pregnancy + Admission ] | 30 |
| 127 | (audit or audits or audited or auditted or auditing or auditings).ti,ab. | 18 |
| 128 | statistic*.ti,ab. | 1640 |
| 129 | (data or datum).ti,ab. | 7696 |
| 130 | national health program*.ti,ab. | 0 |
| 131 | national health service?.ti,ab. | 13 |
| 132 | (government???? adj2 program*).ti,ab. | 1 |
| 133 | provincial program???.ti,ab. | 0 |
| 134 | provincial health*.ti,ab. | 0 |
| 135 | nation-wide.ti,ab. | 0 |
| 136 | state-wide.ti,ab. | 1 |
| 137 | province-wide.ti,ab. | 1 |
| 138 | (nation?? adj3 database?).ti,ab. | 35 |
| 139 | (government?? adj3 database?).ti,ab. | 0 |
| 140 | (state?? adj3 database?).ti,ab. | 0 |
| 141 | (provincial??? adj3 database?).ti,ab. | 0 |
| 142 | ((multi-centre? or multi-center?) adj2 (study or studies)).ti,ab. | 9 |
| 143 | ((multicentre? or multicenter?) adj2 (study or studies)).ti,ab. | 20 |
| 144 | (international adj3 database?).ti,ab. | 22 |
| 145 | (register? or registries).ti,ab. | 6636 |
| 146 | (maternal adj3 audit?).ti,ab. | 0 |
| 147 | (population* adj3 (study or studies)).ti,ab. | 148 |
| 148 | (maternal adj3 surveillanc*).ti,ab. | 0 |
| 149 | (nation?? adj3 surveillance).ti,ab. | 0 |
| 150 | (government* adj3 surveillance).ti,ab. | 0 |
| 151 | (provinc* adj3 surveillance).ti,ab. | 0 |
| 152 | ((state or states) adj3 surveillance).ti,ab. | 0 |
| 153 | (multicountry adj2 survey?).ti,ab. | 0 |
| 154 | (multi-country adj2 survey?).ti,ab. | 0 |
| 155 | or/127-153 [ Data ] | 7711 |
| 156 | 126 and 155 [ ICU + Pregnancy + Admission + Data ] | 30 |
| 157 | Neonatal Intensive Care?.ti. | 3 |
| 158 | NICU.ti. | 0 |
| 159 | NICUs.ti. | 0 |
| 160 | or/157-159 [ Removal of NICU ] | 3 |
| 161 | 156 not 160 | 30 |
| 162 | limit 161 to full systematic reviews | 30 |
