# Supplementary material for: Variability in intensive care unit admission among pregnant and postpartum women in Canada: a nationwide population-based observational study
Source: Crit Care. 2019 Nov 27;23:381. doi: 10.1186/s13054-019-2660-x (PMC6881971; doi:10.1186/s13054-019-2660-x)
Supplement: Supplementary file 13 — Additional file 13: Table S13. General characteristics of maternal death according to whether admitted to ICU or not. [file 13054_2019_2660_MOESM13_ESM.docx]

Table S13. General characteristics of maternal death according to whether admitted to ICU or not

|  | Maternal death with ICU Admission* (n=129) | Maternal death without  ICU Admission*  (n=64) | Standardized difference† | | | |  |  |
| --- | --- | --- | --- | --- | --- | --- | --- | --- |
| Age (years) | 31.5±6.4 | 31.2±7.2 | 0.05 | | | |  |  |
| Maternal comorbidity index | 2.05±2.68 | 1.01±1.62 | 0.47 | | | |  |  |
| Parity |  |  | 0.19 | | | |  |  |
| 0 | 83 (64.3%) | 36 (56.2%) |  | | | |  |  |
| 1 | 28 (21.7%) | 19 (29.7%) |  | | | |  |  |
| 2 + | 18 (13.9%) | 9 (14.0%) |  | | | |  |  |
| Residence rurality |  |  | 0.04 | | | |  |  |
| Urban | 106 (82.8%) | 52 (81.2%) |  | | | |  |  |
| Rural | 22 (17.2%) | 12 (18.7%) |  | | | |  |  |
| Residence income quintile |  |  | 0.26 | | | |  |  |
| Quintile 1 (lowest) | 40 (35.4%) | 14 (25.4%) |  | | | |  |  |
| Quintile 2 | 21 (18.6%) | 15 (27.3%) |  | | | |  |  |
| Quintile 3 | 26 (23.0%) | 13 (23.6%) |  | | | |  |  |
| Quintile 4 | 20 (17.7%) | 10 (18.2%) |  | | | |  |  |
| Quintile 5 (highest) | 6 (5.3%) | -** |  |  |  |  |  |  |
| Hospital rurality |  |  | - 0.18 |  |  |  |  |  |
| Urban | 125 (98.4%) | 64 (100%) |  |  |  |  |  |  |
| Rural | - ** | 0 (0%) |  |  |  |  |  |  |
| Transfer to another institution for acute care | 30 (23.3%) | - ** | 0.49 |  |  |  |  |  |
| Severe maternal morbidity | 87 (67.4%) | 48 (75.0%) | - 0.16 |  |  |  |  |  |

*: Data are presented as mean±SD, median [inter-quartiles] or %.

**: Not reported (cells with 1-5 counts of severe maternal morbidity were suppressed as per privacy policy of the Canadian Institute for Health Information).

†: Standardized difference = difference in means or proportions divided by standard error. Standardized mean differences of 0.2, 0.5, and 0.8 are often/generally equated to effect sizes of small, medium, and large
