# Supplementary material for: Variability in intensive care unit admission among pregnant and postpartum women in Canada: a nationwide population-based observational study
Source: Crit Care. 2019 Nov 27;23:381. doi: 10.1186/s13054-019-2660-x (PMC6881971; doi:10.1186/s13054-019-2660-x)
Supplement: Supplementary file 14 — Additional file 14: Table S14. Estimated odds ratios for multilevel logistic regression model for Intensive Care Unit admission with categorized Maternal Comorbidity Index. [file 13054_2019_2660_MOESM14_ESM.docx]

Table S14. Estimated odds ratios for multilevel logistic regression model for Intensive care unit admission with categorized Maternal Comorbidity Index

| Variable | Odds ratio (95% CI) |
| --- | --- |
| Patient variables |  |
| Maternal Comorbidity Index |  |
| 0 | Reference |
| 1 | 1.90 (1.79-2.02) |
| > 2 | 8.67 (8.24-9.12) |
| Age (years) |  |
| < 15 | 1.79 (0.79-4.04) |
| 15-19 | 1.12 (1.00-1.25) |
| 20-24 | Reference |
| 25-29 | 1.05 (0.98-1.13) |
| 30-34 | 1.19 (1.10-1.28) |
| 35-39 | 1.50 (1.39-1.62) |
| 40-44 | 2.24 (2.02-2.50) |
| > 44 | 2.86 (2.21-3.70) |
| Parity | 0.78 (0.76-0.81) |
| Residence (Urban/rural) | 1.09 (1.02-1.16) |
| Transfer between hospitals | 11.48 (10.74-12.28) |
| Income quintile |  |
| 1 (lowest) | 1.43 (1.32-1.53) |
| 2 | 1.28 (1.19-1.38) |
| 3 | 1.19 (1.10-1.28) |
| 4 | 1.11 (1.03-1.21) |
| 5 (highest) | Reference |
|  |  |
| Hospital variables |  |
| Hospital (Urban versus rural) | 1.30 (0.94-1.79) |
| Groups according to Hospital volume of pregnancy |  |
| 1 (lowest volume) | 2.21 (1.51-3.23) |
| 2 | Reference |
| 3 | 1.35 (0.98-1.85) |
| 4 | 1.51 (1.07-2.04) |
| 5 (highest volume) | 1.32 (0.96-1.81) |
| Province |  |
| Newfoundland and Labrador | 1.24 (0.78-1.96) |
| Prince Edward Island | 0.48 (0.17-1.36) |
| Nova Scotia | 0.71 (0.44-1.16) |
| New Brunswick | 0.64 (0.41-1.02) |
| Ontario | Reference |
| Manitoba | 0.34 (0.22-0.53) |
| Saskatchewan | 0.64 (0.44-0.94) |
| Alberta | 0.35 (0.27-0.46) |
| British Columbia | 0.48 (0.37-0.62) |
| Territories | 0.50 (0.15-1.66) |
