# Supplementary figures and images for: Variability in intensive care unit admission among pregnant and postpartum women in Canada: a nationwide population-based observational study
Source: Crit Care. 2019 Nov 27;23:381. doi: 10.1186/s13054-019-2660-x (PMC6881971; doi:10.1186/s13054-019-2660-x)

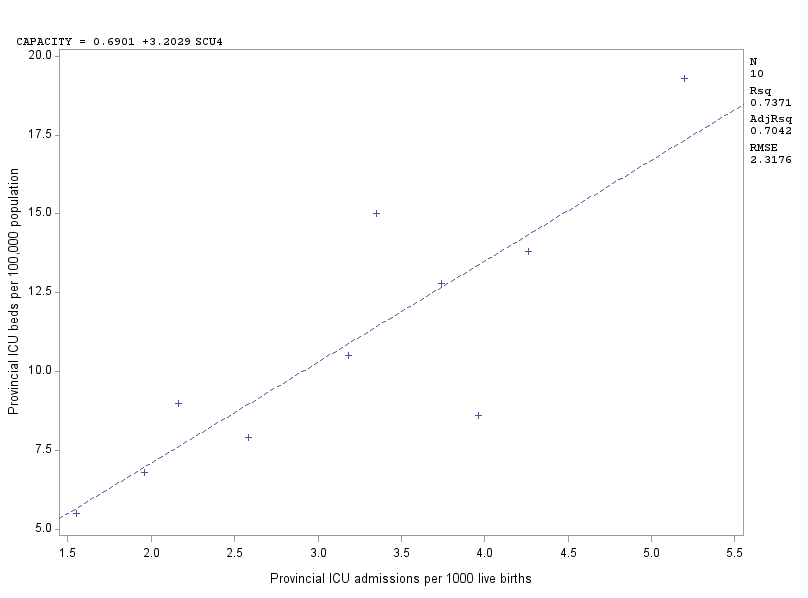

Supplement: Supplementary file 15 — Additional file 15: Figure S1. Provincial ICU Beds per 100,000 population and Provincial Intensive Care Unit Admissions per 1000 Live Births. [file 13054_2019_2660_MOESM15_ESM.png]
